# Associations between DNA methylation and BMI vary by metabolic health status: a potential link to disparate cardiovascular outcomes

**DOI:** 10.1186/s13148-021-01194-3

**Published:** 2021-12-22

**Authors:** Whitney L. Do, Steve Nguyen, Jie Yao, Xiuqing Guo, Eric A. Whitsel, Ellen Demerath, Jerome I. Rotter, Stephen S. Rich, Leslie Lange, Jingzhong Ding, David Van Den Berg, Yongmei Liu, Anne E. Justice, Weihua Guan, Steve Horvath, Themistocles L. Assimes, Parveen Bhatti, Kristina Jordahl, Aladdin Shadyab, Celina I. Valencia, Aryeh D. Stein, Alicia Smith, Lisa R. Staimez, Karen Conneely, K. M. Venkat Narayan

**Affiliations:** 1 Nutrition and Health Sciences Program, Laney Graduate School, Emory University, 1518 Clifton Rd, Atlanta, GA 30322 USA; 2 Herbert Wertheim School of Public Health and Human Longevity Science, University of California, San Diego, CA, USA; 3 Department of Pediatrics, The Institute for Translational Genomics and Population Sciences, The Lundquist Institute for Biomedical Innovation at Harbor-UCLA Medical Center, Torrance, CA USA; 4 Departments of Epidemiology and Medicine, University of North Carolina, Chapel Hill, NC USA; 5 Division of Epidemiology and Community Health, School of Public Health, University of Minnesota, Minneapolis, MN USA; 6 Center for Public Health Genomics, University of Virginia, Charlottesville, VA USA; 7 Division of Biomedical Informatics & Personalized Medicine, School of Medicine, Colorado University Anschutz Medical Campus, Aurora, CO USA; 8 Gerontology and Geriatric Medicine, School of Medicine, Wake Forest, Winston-Salem, NC USA; 9 Department of Population and Public Health Sciences, University of Southern California, Los Angeles, CA USA; 10 Duke Molecular Physiology Institute, Duke University, Durham, NC USA; 11 Center for Biomedical and Translational Informatics, Geisinger, Wilkes-Barre, PA USA; 12 Division of Biostatistics, School of Public Health, University of Minnesota, Minneapolis, MN USA; 13 Department of Human Genetics, University of California, Los Angeles, Los Angeles, CA USA; 14 Department of Medicine, Stanford University, Stanford, CA USA; 15 Cancer Control Research, BC Cancer, Vancouver, BC Canada; 16 Department of Epidemiology, University of Washington, Seattle, WA USA; 17 College of Medicine, University of Arizona Cancer Center, Tucson, AZ USA; 18 Hubert Department of Global Health, Rollins School of Public Health, Emory University, Atlanta, GA USA; 19 Department of Gynecology and Obstetrics, School of Medicine, Emory University, Atlanta, GA USA; 20 Department of Human Genetics, Emory University, Atlanta, GA USA

**Keywords:** DNA methylation, Metabolically healthy, Obesity, Epigenetics

## Abstract

**Background:**

Body mass index (BMI), a well-known risk factor for poor cardiovascular outcomes, is associated with differential DNA methylation (DNAm). Similarly, metabolic health has also been associated with changes in DNAm. It is unclear how overall metabolic health outside of BMI may modify the relationship between BMI and methylation profiles, and what consequences this may have on downstream cardiovascular disease. The purpose of this study was to identify cytosine-phosphate-guanine (CpG) sites at which the association between BMI and DNAm could be modified by overall metabolic health.

**Results:**

The discovery study population was derived from three Women’s Health Initiative (WHI) ancillary studies (*n* = 3977) and two Atherosclerosis Risk in Communities (ARIC) ancillary studies (*n* = 3520). Findings were validated in the Multi-Ethnic Study of Atherosclerosis (MESA) cohort (*n* = 1200). Generalized linear models regressed methylation *β* values on the interaction between BMI and metabolic health *Z* score (BMI × MHZ) adjusted for BMI, MHZ, cell composition, chip number and location, study characteristics, top three ancestry principal components, smoking, age, ethnicity (WHI), and sex (ARIC). Among the 429,566 sites examined, differential associations between BMI × MHZ and DNAm were identified at 22 CpG sites (FDR *q* < 0.05), with one site replicated in MESA (cg18989722, in the TRAPPC9 gene). Three of the 22 sites were associated with incident coronary heart disease (CHD) in WHI. For each 0.01 unit increase in DNAm *β* value, the risk of incident CHD increased by 9% in one site and decreased by 6–10% in two sites over 25 years.

**Conclusions:**

Differential associations between DNAm and BMI by MHZ were identified at 22 sites, one of which was validated (cg18989722) and three of which were predictive of incident CHD. These sites are located in several genes related to NF-kappa-B signaling, suggesting a potential role for inflammation between DNA methylation and BMI-associated metabolic health.

**Supplementary Information:**

The online version contains supplementary material available at 10.1186/s13148-021-01194-3.

## Background

Obesity rates continue to rise with obesity occurring in more than 41.1% of women in the USA in 2016 [1]. While obesity is most typically defined as body mass index (BMI) > 30 kg/m^2^, limitations in the use of BMI have been noted, including variation in associations with health outcomes by race/ancestry, physical activity, and age [2, 3], as well as some reports finding no association between higher-risk categories (overweight and middle obesity) with mortality [4, 5]. These conflicting reports have motivated several studies to examine whether differential phenotypes of obesity exist and whether examining BMI in isolation of additional metabolic health parameters is a sufficient metric of overall health.

A growing body of evidence has found heterogeneity in obesity, with some phenotypes exhibiting differential risk for cardiovascular outcomes. Metabolically healthy obesity (MHO) has been defined as obesity with less than two or three metabolic health risk factors. Some but not all studies have found MHO to be associated with reduced risk of cardiovascular outcomes compared to metabolically unhealthy obesity (MUO) [6–11]. In a recent systematic review, MHO had a higher risk of cardiovascular events than metabolically healthy, normal weight participants (risk ratio [RR] 1.45, 95% CI 1.20–1.70, reference metabolically healthy, normal weight), but had lower risk to metabolically unhealthy normal weight (RR 2.07, 95% CI 1.62–2.65, reference metabolically healthy, normal weight) and MUO individuals (RR 2.31, 95% CI 1.99–2.69, reference metabolically healthy, normal weight) [12]. These findings suggest that metabolic health status may differentially influence the relationship between BMI and health outcomes. Examining the molecular underpinnings of this phenotype may guide our understanding of this epidemiological phenomenon by identifying the biological mechanisms which may be leading to a reduction in risk of health outcomes associated with obesity. Additionally, identifying biomarkers of MHO, particularly if they can identify individuals more likely to remain in MHO, would be advantageous for more targeted interventions.

**Fig. 1:**
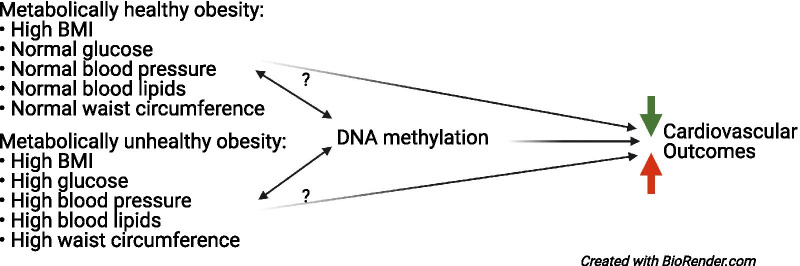
Conceptual framework research question. Abbreviations: BMI, body mass index

Epigenetic mechanisms, such as DNA methylation (DNAm), are important biological features to examine in the context of chronic diseases such as obesity and metabolic health. Changes to DNAm can induce changes in gene expression in causal disease pathways potentially mediating or modifying differential health outcomes [13]. Obesity has been widely examined and shown to associate with prolific methylation changes in the blood and adipose tissue [14–16]. Similarly, metabolic syndrome and metabolic health risk factors have been found to associate with differential methylation [17–21]. Indeed the mouse model which is used to represent MHO is developed from deletion of the *BRD2* gene, which is a primary epigenetic regulator of histone acetylation [22]. However, no studies have integrated these phenotypes to examine how BMI-associated methylation varies by metabolic health status. Particularly since DNAm has been reported to mediate the relationship between obesity and increased cardiovascular outcomes [23], evaluating the epigenome may provide insight into pathways contributing to the differences in outcomes. Thus, the purpose of this study is to examine whether BMI associates with methylation differentially according to metabolic health status (Fig. 1).

## Results

**Fig. 2:**
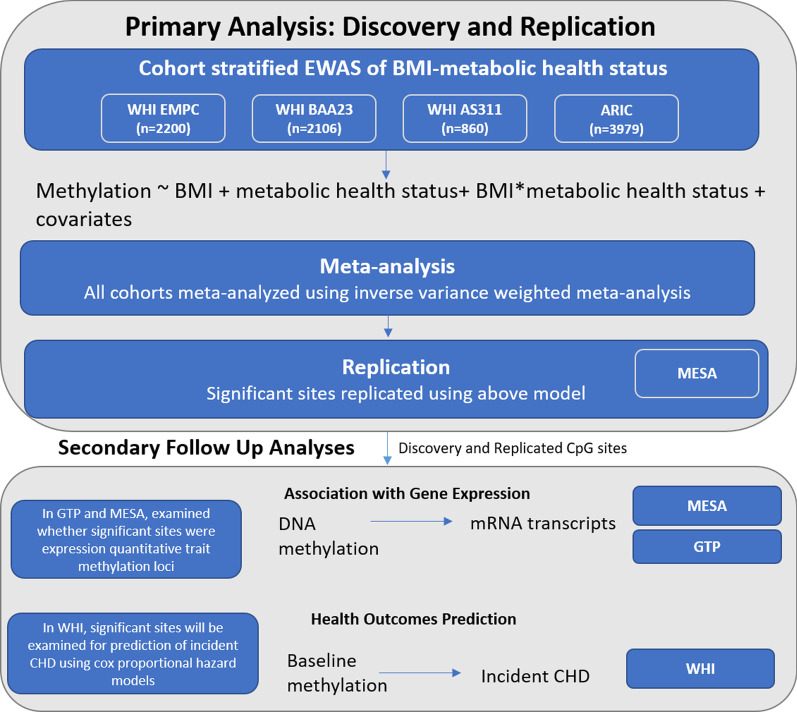
Summary of the analyses. Abbreviations: WHI, Women’s Health Initiative; EMPC, Epigenetic Mechanisms of Particulate Matter-Mediated Cardiovascular Disease; BAA23, the Integrative Genomics for Risk of Coronary Heart Disease and Related Phenotypes in WHI cohort; AS311, Bladder Cancer and Leukocyte Methylation; ARIC, Atherosclerosis Risk in Communities study; BMI, body mass index; CHD, coronary heart disease; MESA, Multi-Ethnic Study of Atherosclerosis; GTP, Grady Trauma Project

A summary of the methods is included in Fig. 2.

**Fig. 3:**
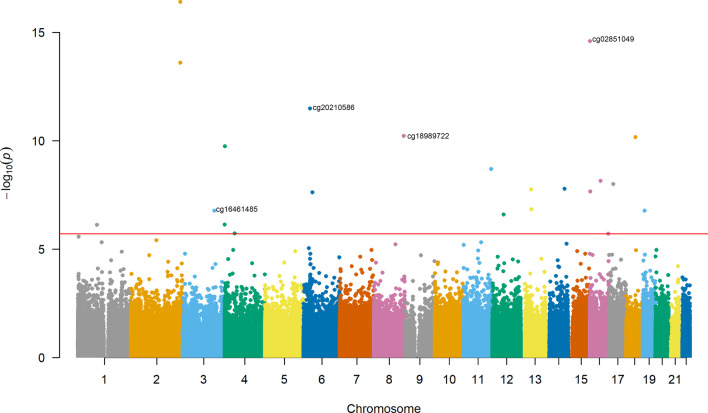
Manhattan plot of the association between the interaction of BMI and metabolic health *Z* score and DNA methylation. Significant sites identified as those above the red line (FDR < .05; *p* < 2 × 10^–6^)

Demographic characteristics of the cohorts are described in Table 1 for the three ancillary studies from the Women’s Health Initiative (WHI) including *Epigenetic Mechanisms of Particulate Matter-Mediated Cardiovascular Disease* (EMPC, aka AS315), the *Integrative Genomics for Risk of Coronary Heart Disease and Related Phenotypes in WHI cohort* (BAA23), and *Bladder Cancer and Leukocyte Methylation* (AS311) and the two ancillary studies from the Atherosclerosis Risk in Communities study (ARIC) including European Americans (EA) and African Americans (AA). Overall, 7497 participants were included in the discovery analysis. To examine the differential impact of metabolic health status on BMI, linear regression models were used regressing the methylation *β* value on the interaction term for BMI and metabolic health status, adjusting for each higher-level variable (BMI and metabolic health) and covariates. We conducted two epigenome-wide assocation study (EWAS) with metabolic health status defined dichotomously (BMI × MH) and continuously (BMI x metabolic health Z-score [MHZ]). We identified no statistically significant differential associations between cytosine and guanine nucleotide pair (CpG) methylation and BMI by dichotomized metabolic health status (BMIxMH). When metabolic health status was examined continuously (MHZ), 22 CpG sites were associated with BMIxMHZ (false discovery rate [FDR] *q* value < 0.05, Table 2; Fig. 3). For ease of interpretation, we described the direction of effect in the 22 significant sites in the models examining BMI × MH  (Additional file 1: Table S1). In 13 of the 22 sites, an increase in BMI was associated with an opposite direction of effect in the coefficient in metabolically healthy vs unhealthy individuals. In the replication analysis in the Multi-Ethnic Study of Atherosclerosis (MESA), cg18989722 associated with BMIxMHZ (*p* < 0.05 in a consistent direction, Additional file 1: Table S2). cg18989722 inversely associated with BMI × MHZ. When examining BMI × MH in this site, every unit increase in BMI was associated with increased DNAm in metabolically healthy individuals and decreased methylation in metabolically unhealthy individuals. We examined a gene ontology analysis of the 22 significant sites. However, no pathways were significantly enriched.

**Table 1:** Demographic characteristics of each ancillary study in the Women’s Health Initiative (WHI) and the Atherosclerosis Risk in Communities (ARIC)

	EMPC (*n* = 1833)	BAA23 (*n* = 1977)	AS311 (*n* = 167)	ARIC EA (*n* = 1059)	ARIC AA (*n* = 2461)
Clinical trial participant					
Yes	1833	1543	119	–	–
No	0	434	48	–	–
Case/control status					
Case	–	987	91	–	–
Control	–	990	76	–	–
Age mean (SD)	63.2 (7.1)	64.6 (7.1)	66.2 (7.2)	59.9 (5.4)	56.6 (5.9)
Sex					
Female	1833	1977	167	611	1574
Male	0	0	0	448	887
Ethnicity					
White	922	944	99	1059	0
African American	474	631	49	0	2461
Hispanic/Latino	260	402	16	0	0
Asian or Pacific Islander	107	0	2	0	0
American Indian or Alaskan Native	41	0	1	0	0
Other	29	0	0	0	0
Smoking status					
Former and current	853	913	99	610	1351
Never	963	1048	67	449	1110
Metabolic health status					
Metabolically healthy	1254	1163	109	662	1177
Metabolically unhealthy	579	814	58	397	1284
BMI*Metabolic Health Z *score mean (SD)*	2.96 (30.38)	2.92 (31.59)	2.84 (30.4)	2.55 (29.0)	2.82 (31.4)
BMI mean (SD)	29.5 (5.9)	29.8 (6.1)	29.3 (6.9)	26.2 (4.3)	30.1 (6.2)
BMI categories					
Underweight	7	13	0	20	18
Normal	417	420	41	435	444
Overweight	641	680	67	429	912
Obese	768	864	59	175	1087
Waist circumference mean (SD)	89.5 (13.8)	90.7 (13.7)	89.1 (15.1)	94.5 (12.8)	101.4 (15.1)
Triglycerides mean (SD)	153 (88.2)	146.9 (83.4)	143.8 (82.5)	140.4 (83.6)	117.3 (77.7)
HDL-cholesterol mean (SD)	58 (15.1)	52.1 (13.2)	53.2 (13.0)	52.3 (18.2)	53.3 (17.3)
Systolic blood pressure mean (SD)	128 (18)	132.1 (17.8)	132.5 (16.6)	118.7 (18.0)	127.3 (20.6)
Diastolic Blood Pressure mean (SD)	75.3 (9.4)	76.4 (9.3)	76.3 (8.5)	68.5 (9.7)	75.2 (10.7)
Blood glucose mean (SD)	103 (31.1)	108.6 (41.3)	105.2 (37.2)	105.9 (28.6)	129.3 (64.3)

Means [standard deviation (SD)] or proportions have been included

EMPC, epigenetic mechanisms of particulate matter-mediated cardiovascular disease; BAA23, the integrative genomics for risk of coronary heart disease and related phenotypes in WHI cohort; AS311, bladder cancer and leukocyte methylation; BMI, Body Mass Index; EA, European American; AA, African American

**Table 2:** Significant sites associated with body mass index * metabolic health *Z* score interaction (BMIxMHZ) including the effect size for the higher-level variables

CpG site	Effect size (BMI × MHZ)	Standard error	*Z* score	*P* value	FDR *q* value	Direction	Effect size (BMI)	Effect size (MHZ)
cg24827562	− 8.71E−05	9.83E−06	− 8.41E+00	3.98E−17	1.70E−11	−+++−	9.27E−06	0.00161952
cg02851049	− 8.85E−05	1.04E−05	− 7.91E+00	2.53E−15	5.39E−10	++−+−	− 8.49E−06	0.00126491
cg22076143	− 1.01E−04	1.27E−05	− 7.62E+00	2.55E−14	3.63E−09	−+++−	− 1.47E−06	0.00119747
cg20210586	− 1.03E−04	1.39E−05	− 6.97E+00	3.28E−12	3.49E−07	−−++−	1.38E−05	0.00237264
cg18989722	− 8.36E−05	1.20E−05	− 6.54E+00	6.14E−11	4.86E−06	−+−+−	− 5.73E−06	0.00139622
cg15062225	− 1.38E−04	2.05E−05	− 6.52E+00	6.83E−11	4.86E−06	−+−+−	8.86E−05	0.00309348
cg24460625	− 7.26E−05	1.11E−05	− 6.38E+00	1.80E−10	1.10E−05	−+−+−	2.29E−06	0.00186604
cg10057841	− 9.86E−05	1.56E−05	− 5.99E+00	2.04E−09	1.09E−04	−−−+−	3.25E−05	0.0020491
cg06344952	− 9.11E−05	1.46E−05	− 5.79E+00	7.23E−09	3.43E−04	−−−+−	5.91E−05	0.00307032
cg26206680	− 6.13E−05	9.90E−06	− 5.73E+00	9.98E−09	4.26E−04	++−+−	− 7.74E−06	0.00014127
cg27004639	5.20E−05	9.14E−06	5.64E+00	1.66E−08	6.34E−04	+−+−+	4.21E−05	− 0.0017364
cg19572849	− 4.99E−05	8.96E−06	− 5.63E+00	1.78E−08	6.34E−04	−+++−	− 1.67E−05	0.00155317
cg08082299	− 1.01E−04	1.73E−05	− 5.59E+00	2.25E−08	7.37E−04	−+++−	− 1.49E−05	0.00209263
cg18298785	− 8.52E−05	1.40E−05	− 5.58E+00	2.42E−08	7.37E−04	−+++−	− 2.99E−07	0.00212176
cg16543390	6.71E−05	1.38E−05	5.26E+00	1.46E−07	4.16E−03	−+−++	3.05E−05	− 0.0022199
cg16461485	− 1.15E−04	2.06E−05	− 5.23E+00	1.71E−07	4.34E−03	−+−+−	8.27E-05	0.00151377
cg21880445	− 7.90E−05	1.44E−05	− 5.23E+00	1.73E−07	4.34E−03	−+++−	− 3.03E−05	0.00174726
cg07226317	− 9.17E−05	1.62E−05	− 5.16E+00	2.53E−07	6.00E−03	−+−+−	3.12E−05	0.00135378
cg05441596	− 5.74E−05	1.11E−05	− 4.95E+00	7.39E−07	1.62E−02	++++−	3.48E−05	0.00106747
cg24720717	6.22E−05	1.22E−05	4.95E+00	7.59E−07	1.62E−02	++−−+	− 7.25E−06	− 0.002551
cg11553983	− 6.96E−05	1.36E−05	− 4.76E+00	1.94E−06	3.88E−02	+−−+−	3.50E−05	0.00075824
cg00868074	3.15E−04	6.90E−05	4.75E+00	2.00E−06	3.88E−02	+++++	− 0.0003756	− 0.0140558

Direction of effect has been included for the individual ancillary studies from the WHI, Women’s Health Initiative; EMPC, epigenetic mechanisms of particulate matter-mediated cardiovascular disease; BAA23, the integrative genomics for risk of coronary heart disease and related phenotypes in WHI cohort; AS311, bladder cancer and leukocyte methylation; ARIC, the Atherosclerosis Risk in Communities Study; EA, European American; AA, African American, respectively

**Fig. 4:**
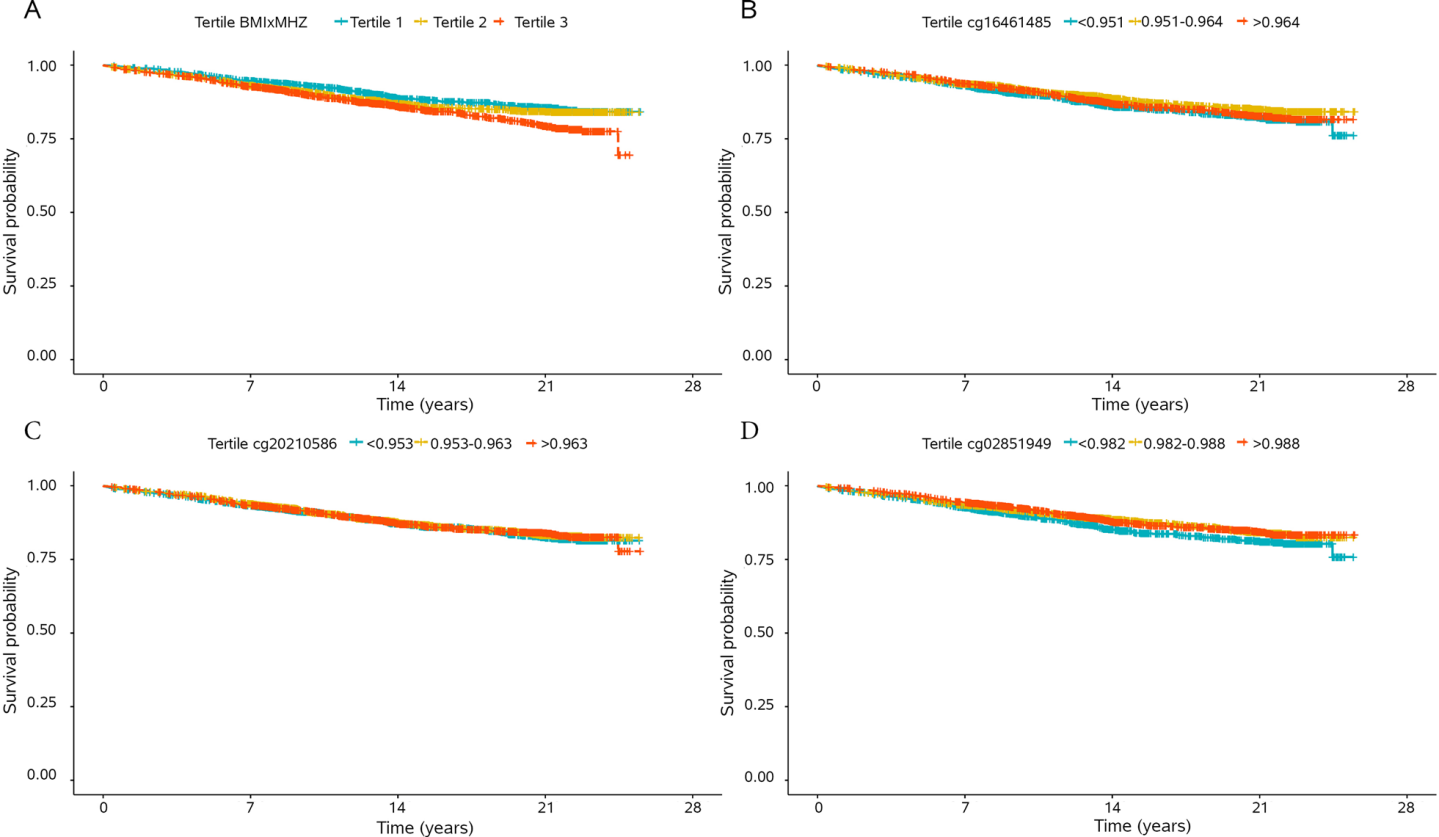
Probability of incident CHD by tertile of the interaction between body mass index (BMI) and metabolic health *Z* score (BMI × MHZ) (**A**) and CpG site methylation of cg16461485 (**B**), cg20210586 (**C**), and cg02851049 (**D**) over 25 years in the Women’s Health Initiative (WHI)

Given the known relationships between obesity and metabolic status and cardiovascular disease, we examined whether DNAm taken at baseline predicted incident myocardial infarction over 25 years in the WHI. After excluding individuals from WHI with a history of cardiovascular disease, 3746 individuals remained (BAA23 *n* = 1823, EMPC *n* = 1775, AS311 *n* = 148). In WHI, there were 714 events (number of events = 585 [BAA23], 113 [EMPC], 16 [AS311]) with an average follow up of 14–15 years (mean follow up = BAA23 14.0 years, EMPC 15.69 years, AS311 15.85 years). When predicting incident coronary heart disease (CHD), we initially examined whether the interaction between BMI × MHZ taken at baseline was associated with incident CHD, adjusting for BMI, MHZ, case–control status, age, smoking status, and ethnicity. BMIxMHZ was significantly associated with incident CHD (hazard ratio [HR] 1.02, 95% CI 1.004, 1.03, *p *value = 0.005, Fig. 4A). Then we added the DNA methylation at cg18989722 as a predictor and found that it was not associated with incident CHD (Additional file 1: Table S3). However, when examining the 22 sites from the discovery analysis, three sites were associated with incident CHD adjusted for the reduced set of covariates (*p* < 0.05, Fig. 4B–D; Table 3). In models adjusting for the full set of covariates, two sites were significantly associated with CHD, cg16461485 and cg16543390.

**Table 3:** Significant CpG sites associated with incident coronary heart disease (CHD) in the Women’s Health Initiative (WHI) over 25 years

CpG site	Hazard ratio	95% CI	*P* value
cg02851049^a^	0.90	(0.81, 0.99)	0.030
cg20210586^a^	1.09	(1.00, 1.19)	0.046
cg16461485^a^	0.94	(0.89, 0.99)	0.031
cg16461485^b^	0.93	(0.88, 0.99)	0.023
cg16543390^b^	1.06	(1.01, 1.11)	0.028

Cox proportional hazard model examining the association between incident CHD and *β* value of the 22 CpG sites in discovery analysis. Models adjusted for age, race/ethnicity, smoking status, case–control status (BAA23 and AS311), DNA methylation array, row, and cell composition in reduced model^a^ and reduced model covariates and physical activity and diet in the full model^b^

For the replicated site and the three sites associated with incident CHD in the reduced set of covariates, we examined whether they were associated with differential gene expression in the blood in two cohorts: Grady Trauma Project (GTP) and MESA (Additional file 1: Table S4). None of the sites were reported to be associated with gene expression in *cis*. cg18989722 (in chromosome 8) was associated with differential expression of *PTGS1* in chromosome 9 and cg16461485 (in chromosome 3) was associated with differential expression of *TNFRSF13B* in chromosome 17 representing *trans* associations.

**Table 4:** Adult treatment panel (ATP) III clinical identification of metabolic syndrome

Clinical measure	Defining level
Waist circumference	≥ 102 cm in men or ≥ 88 cm in women
Triglycerides	≥ 150 mg/dL or drug treatment for elevated triglycerides
High density lipoprotein (HDL)	< 40 mg/dL in men or < 50 mg/dL in women or drug treatment for reduced HDL
Blood pressure	≥ 130/85 mmHG or drug treatment for hypertension
Glucose	≥ 110 mg/dL or drug treatment for elevated glucose

In sensitivity analyses, we examined the change in the association of BMI × MHZ with the 22 significant CpG sites in the WHI and ARIC when adjusted for physical activity. After including physical activity as a covariate, BMI × MHZ was no longer significantly associated with any CpG site. However, this may be in part due to a reduction in power as the effect sizes did not change significantly in the significant sites (correlation in effect sizes from main analysis compared to analysis adjusted for physical activity = 0.92). When examining the change in the effect size when sequentially leaving out each of the metabolic health parameters one at a time from the MHZ score, omission of high-density lipoprotein cholesterol (HDL) most potently attenuated the effect estimate (effect estimate correlation = 0.89, Additional file 1: Table S5). In the 22 sites, effect sizes and *Z* scores changed minimally when adjusted for lipid, hypertension, and glycemic medications (all correlations in effect size = 0.97 and all correlations in *Z* scores = 0.95). We examined the influence of individual ancillary studies on the results by examining the change in significance and effect size when ancillary studies (BAA23, EMPC, AS311, ARIC AA, and ARIC EA) were individually excluded from the analysis. Significance changed moderately with exclusion of each study with 30, 20, 25, 20 and 27 significant sites when BAA23, EMPC, AS311, ARIC AA, and ARIC EA were excluded, respectively. Differences in effect size were minor (correlation with main analysis = 0.99) in all studies except with exclusion of ARIC AA (correlation with main analysis = 0.86, Additional file 1: Figures S1–S5). We additionally examined the residuals of the four sites described above in the EWAS models to assess violations of a non-normal distribution (Additional file 1: Figures S6–S13). These plots appear to be consistent with a normal distribution, with the exception of ARIC EA. When ARIC EA is excluded from the analysis, all 22 remain significant and *Z* scores tend to be higher as compared to the full analysis.

## Discussion

In this study, we found 22 CpG sites were associated with BMIxMHZ in the WHI and ARIC cohorts, with one site replicating in a consistent direction in MESA. Among the 22 sites, two CpG sites inversely and one CpG site positively associated with a change in incidence of CHD over 25 years in the WHI cohort.

One site replicated in MESA in a consistent direction, cg18989722 located in the body of the *TRAPPC9* gene. *TRAPPC9* has been shown to play a role in NF-kappa-B signaling by activating NF-kappa-B through increased phosphorylation of the IKK complex [24]. *TRAPPC9* encodes NIBP, which binds to IKK/NIK to enhance NF-kappa-B activation. *TRAPPC9* has recently been identified as an imprinted gene primarily expressing the maternal allele (70% of transcripts in the brain expressed maternal allele). *TRAPPC9* knock-out mice exhibit a rare intellectual disability accompanied by an increase in fat mass and body weight [25], suggesting that expression of this gene may protect against obesity. Several CpG sites in *TRAPPC9* have been identified in EWAS of childhood adiposity [26, 27]. As gene body methylation has often been cited as an indicator of an active gene [28], our findings are in alignment with previous reports of protection against obesity since individuals with lower BMIxMHZ had higher methylation in this site.

This site was also associated with increased gene expression of the *PTGS1* gene. *PTGS1* (also known as *COX1*) catalyzes the conversion of arachinodate to prostaglandin protein and is inhibited by anti-inflammatory drugs. In our sensitivity analysis, when adjusted for lipid medication use including peripheral vasodilators such as aspirin, the effect size moderately changed (− 8.36 × 10^–5^ in unadjusted models and − 5.25 × 10^–5^ in adjusted models). However, the *Z* score was smaller (− 6.54 in unadjusted models and − 2.55 in adjusted models). This suggests some attenuation in the relationship between BMI × MHZ and DNAm in this site is potentially modified by medication use.

Methylation in three sites was associated with incident CHD over 25 years in the WHI cohort: cg16461485 located in the body of *SELT*, cg02851049 located in the body of *POLR3K*, and cg20210586 in the body of *TRIM39.* None of these sites have been identified in previous EWAS. We also found that cg16461485 associated with reduced gene expression of *TNFRSF13B*, which encodes the tumor necrosis factor (TNF) receptor superfamily member 13B, also known as the transmembrane activator and CAML interactor (TACI). This protein activates NFAT, AP1, and NF-kappa-B [29]. TACI knock-out mice were protected against high-fat-diet-induced inflammation and dysglycemia, which may be mediated by a shift in adipose tissue macrophages from M1 to M2, which tend to promote a phenotype of insulin sensitivity [30]. These findings further support the role that methylation in cg16461485 exhibiting a protective effect.

Given the molecular functions of these genes, differential inflammatory mechanisms (potentially mediated by the NF-kappa-B pathway) may account for the observed differences in health outcomes by BMIxMHZ. Obesity and several of the metabolic health parameters associate with excess inflammation [31]. However, some studies have posited that MHO may be due to an uncoupling of obesity and insulin resistance due to differential inflammatory mechanisms as MHO has been associated with lower inflammatory markers including C-reactive protein (CRP), TNF-α, interleukin-6 (IL-6), and plasminogen activator inhibitor-1 [32, 33]. As the CpG sites identified in this study were adjusted for cell composition which uses surrogate measures from six cell types [34], we may be identifying unique immune cell subsets associated with these disease exposures not captured by this method which may drive differences in outcomes. Additionally, several studies have also observed a unique relationship between inflammatory markers and adiposity in individuals of African descent, where these markers do not appear to be as sensitive to adiposity compared to individuals of European descent [35, 36]. This may explain the significant differences observed when we exclude the ARIC AA cohort in sensitivity analyses.

When we adjusted for physical activity, no sites were significantly associated with BMI × MHZ. We included this analysis as several studies have touted that MHO may be the product of increased fitness in this population [36, 37]. This may be due to true confounding by physical activity. However, there may also be collinearity, as physical activity is highly associated with BMI (*p* = 2.5e−17) and metabolic health (*p* = 2.94e−12), which would lead to a reduction in significance in the identified variables. Future studies could further explore these associations in physical activity interventions in these populations.

There are several important limitations in this study. Given the cross-sectional design, we cannot determine any causal association and may be at risk of reverse causality, if methylation is contributing to changes in BMI or metabolic risk factors. Moreover, metabolic risk factors may also be a product of duration of obesity, since several studies have found MHO to be a transitory state [6, 7, 37]. However, understanding the methylomic differences in these populations would still be advantageous to identify biological mechanisms that may be driving the differences in outcomes. Another limitation includes the potential for confounding by cell composition. While we found unique relationships between three CpG sites and CHD, none of these sites replicated in an external population suggesting that other confounding factors may be causing this association. Nevertheless, the limited replication may be due in part to limited power as the replication analysis had the power to detect effect sizes as low as 0.01 and the effect sizes from our discovery EWAS were much lower (Additional file 1: Table S6). Additionally, the results may have diverged between ARIC and WHI populations since the WHI includes only women. While we adjusted for sex in ARIC, we may be identifying signals in WHI that are differential in women versus men. A strength of this study is examining the unique interaction between BMI and metabolic health in three population-based cohort studies and examining their impact on gene expression and CHD outcomes.

Overall, we found four CpG sites which may have a unique relationship with BMI in metabolically healthy vs unhealthy individuals. Our study findings may align with several studies suggesting that differential inflammatory mechanisms may account for differences in metabolic risk factors associated with increasing BMI. Future research studies could benefit from examining longitudinal changes in methylation associated with change in metabolic health status to determine the direction of effect and single cell epigenomic signatures of obesity and metabolic health to examine how individual cell profiles influence this relationship.

## Methods

### Study population

Two cohorts were used in the discovery phase: the WHI and the ARIC. Data from three WHI ancillary studies were included: EMPC, aka AS315, the BAA23, and AS311. EMPC assessed epigenetic mechanisms underlying associations between ambient particulate matter air pollution and cardiovascular disease within the WHI Clinical Trials (CT, *n* = 2200). BAA23 was a case–control study assessing predictors of coronary heart disease (CHD) within the WHI CT (*n* = 1664) and observational study (OS, *n* = 442), where cases were identified using eight biomarkers of CHD. AS311 is a matched case–control study of bladder cancer among women within the WHI CT (*n* = 405) and OS (*n* = 455) [38].

ARIC included data from two ancillary studies of AA and EA. ARIC is an ongoing prospective cohort study investigating the etiology of CHD in four US communities: Forsyth County, NC; Jackson, MS; Minneapolis, MN; Washington County; MD. Participants were aged 45–64 and followed up in each community over 30 years with 7 study visits [39, 40]. DNAm was measured in 2879 AA and 1100 EA participants from ARIC in visit 2 (1990–1992) or visit 3 (1993–1995).

The replication cohort derived from the MESA study. MESA is a longitudinal, population cohort study designed to examine risk factors for and the progression of CHD. Participants aged 45–84 years without clinically apparent CHD were recruited between July 2000 and August 2002 from six regions in the USA: Winston-Salem, NC; Northern New York, NY; Baltimore, MD; St. Paul, MN; Chicago, IL; and Los Angeles, CA. DNAm was derived from peripheral blood mononuclear cell samples at Exam 1 or Exam 5 in a random sample of 1200 non-Hispanic white, AA, Hispanic, and Chinese American participants [41, 42].

### Measurements

In WHI, weight, height, waist circumference, and blood pressure (BP) were measured at the physical exam. In ARIC, these measurements were taken at Visit 2 or 3. BMI was calculated as weight (kg)/height (m)^2^. Waist circumference was measured to the nearest 0.5 cm. Systolic/diastolic BP was measured twice and three times in WHI and ARIC, respectively, with the average of the two (WHI) or last two (ARIC) measurements used. Biochemical measurements were analyzed in blood samples collected after a 12-h fast. These include triglycerides (TG), HDL, and fasting glucose.

### Metabolic health exposures

Metabolic health was examined in two ways, dichotomously and continuously. Metabolic risk was dichotomously defined by the presence of three or more components of metabolic syndrome using the Adult Treatment Panel III (ATP III) criteria (Table 4). Thus, MUO and MHO referred to the presence of three or more and less than three components, respectively. Metabolic health was also examined continuously as a *Z* score of the clinical measures used in the ATP III criteria. For each metabolic parameter, for example TG, the *Z* score for TG was created by (TG − mean TG)/standard deviation (TG) of the population. Then all the clinical parameter *Z* scores were pooled to define a MHZ. For HDL, the inverse of HDL was used as a higher MHZ is indicative of poorer health. We examined the HDL variable for normality (Additional file 1: Figure S14–S15). We examined BMI continuously. Individuals were excluded if metabolic health parameters and DNAm were not measured within the same year.

### Covariates

Age, race/ethnicity (White, AA, Hispanic/Latino, Asian, American Indian, other in WHI and EA and AA in ARIC), and smoking status (current/former or never) were self-reported. Physical activity was measured by the Baecke questionnaire in ARIC [43] and a self-administered questionnaire in WHI [44] and expressed as total energy expended from light, moderate, or vigorous intensity recreational physical activity which includes walking, mild, moderate, and strenuous physical activity in kcal/week/kg (MET-hours/week).

### DNA methylation

In the all cohorts, DNA was extracted from peripheral blood leucocytes collected at visit-specific fasting blood draws [45]. In the WHI and ARIC cohorts, DNAm was measured using the Illumina HM450K Infinium Methylation BeadChip. In the MESA cohort, DNAm was measured via the Illumina MethylationEPIC BeadChip array. DNAm was estimated as the proportion of methylated beads relative to combined unmethylated and methylated beads for a specific CpG site defined as the *β *value (ranging from 0 [unmethylated] to 1 [methylated]). All methylation data were normalized using beta-mixture quantile normalization [46]. Technical covariates included chip and row to adjust for batch effects and cell composition, which was estimated using the reference-based Houseman method [34]. Additional quality control procedures for each of the studies has been included in the Additional file 1: Methods. After quality control, 428,278 probes remained in all cohorts and were examined.

### Statistical analysis

We used R (https://www.r-project.org/) for all analyses. We calculated means and standard deviations or counts and proportions for study population characteristics. In the EWAS, all models were stratified by cohort (EMPC, BAA23, AS311 in WHI) or race (AA and EA in ARIC) and pooled using inverse-variance weighted fixed effect meta-analysis. BMI was examined continuously. To examine the differential impact of metabolic health status on BMI, linear regression models were used regressing the methylation β value on the interaction term for BMI and metabolic health status, adjusting for each higher-level variable (BMI and metabolic health) and covariates. We conducted two EWAS with metabolic health status defined dichotomously (BMIxMH) and continuously (BMIxMHZ). Covariates in all models included cell composition, the top three principal components of genetic relatedness, race/ethnicity (WHI), sex (ARIC), smoking status, row, and age. WHI study-specific covariates included trial study and randomization arm (EMPC, BAA23, AS311) and case–control status (BAA23, AS311). To adjust for batch effects, the DNAm array was included as a random effect for each BeadChip in our model. Significant CpG sites were identified by the interaction *p *value at a FDR *q *value < 0.05. We examined the correlation between BMI and MHZ to test for collinearity (Additional file 1: Figure S16). These variables were not highly correlated.

Results identified in the discovery cohorts were replicated in the MESA cohort using linear regression models as previously described. Significant CpG sites were examined using the same linear regression model as above examining BMI × MHZ. Models were adjusted for DNAm array number and row location, cell composition, principal components of genetic relatedness, race/ethnicity, age, sex, alcohol consumption, and smoking. Significant replication was defined at *p* < 0.05 and a consistent direction of effect.

### Outcomes analyses

In significant sites identified through EWAS, DNAm at CpG sites was examined as a predictor of incident CHD in the WHI. CHD was defined by incident myocardial infarction or CHD death. Acute, hospitalized myocardial infarction was identified in medical records on the basis of cardiac pain, electrocardiogram, and biomarker data, and then physician-adjudicated. Further details regarding the review, classification, and adjudication of CHD in WHI have been described [47].

Multivariate Cox proportional hazard ratios were used to examine whether significant sites identified through EWAS (exposure) were associated with incident CHD in WHI. Individuals with a history of (or incident) myocardial infarction or coronary revascularization (angioplasty; stent; bypass) before measurement of DNAm were excluded. Covariates included age, race/ethnicity, smoking status, case–control status (BAA23 and AS311), DNAm array, row, and cell composition in the reduced model. In the full model, we adjusted for the covariates in the reduced model as well as physical activity and diet quality. Diet quality was measured using the Alternative Healthy Eating Index-2010 score [48] derived from food frequency questionnaires in the WHI. Significant sites were defined by *p* < 0.05.

### Gene expression

To elucidate the potential functional implications of the identified CpG sites, we examined gene expression information using previously published significant gene expression quantitative trait methylation loci (eQTMs) summary statistics in blood from MESA and the GTP [49]. We examined the summary statistics for the four CpG sites identified in validation and in secondary CHD analyses. This population from MESA had minimal overlap with the MESA population examined in replication analyses.

### Sensitivity analyses

As metabolic health status is constructed from several metabolic parameters, differences in methylation may be driven by individual metabolic parameters. To assess the degree that individual metabolic parameters influence methylation at significant sites, we reanalyzed associations between BMI × MHZ status and methylation excluding individual metabolic parameter in the MHZ score and compared the effects to the original estimates obtained through EWAS. For the significant sites, we also examined changes in effect size when adjusting for lipid, hypertension and glycemic medication use. We additionally repeated the primary EWAS analysis adjusting for physical activity. We additionally examined the four sites identified in replication and secondary analyses for normality and heteroskedasticity by examining the QQ plots and residual plots from the discovery analysis.

## Supplementary Information

**Additional file 1.** Supplemental Figures 1-16. Supplemental Tables 1-6.

## Data Availability

The data for all studies are publicly available and more information can be found at the individual cohort websites: WHI (www.whi.org), ARIC (https://sites.cscc.unc.edu/aric/desc), and MESA (www.mesa-nhlbi.org).

## Abbreviations

BMI: Body mass index
DNAm: DNA methylation
CpG: Cytosine-phosphate-guanine
WHI: Women’s Health Initiative
ARIC: Atherosclerosis Risk in Communities
MHZ: Metabolic health *Z* score
ATP: Adult treatment panel
BMI × MHZ: Interaction between body mass index and metabolic health *Z *score
FDR: False discovery rate
CHD: Coronary heart disease
MHO: Metabolically healthy obesity
MUO: Metabolically unhealthy obesity
RR: Risk ratio
EMPC: Epigenetic Mechanisms of Particulate Matter-Mediated Cardiovascular Disease
BAA23: Integrative Genomics for Risk of Coronary Heart Disease and Related Phenotypes in WHI cohort
AS311: Bladder Cancer and Leukocyte Methylation
CT: Clinical trial
OS: Observational study
AA: African American
EA: European American
MESA: Multi-ethnic Study of Atherosclerosis
BP: Blood pressure
TG: Triglycerides
HDL: High-density lipoprotein cholesterol
EWAS: Epigenome-wide association study
eQTM: Expression quantitative trait methylation loci
GTP: Grady Trauma Project
HR: Hazard ratio
TNF: Tumor necrosis factor
TACI: Transmembrane activator and CAML interactor
CRP: C-reactive protein
IL-6: Interleukin 6
