# Supplementary material for: Associations between DNA methylation and BMI vary by metabolic health status: a potential link to disparate cardiovascular outcomes
Source: Clin Epigenetics. 2021 Dec 22;13:230. doi: 10.1186/s13148-021-01194-3 (PMC8697469; doi:10.1186/s13148-021-01194-3)
Supplement: Supplementary file 1 — Additional file 1. Supplemental Figures 1-16. Supplemental Tables 1-6. [file 13148_2021_1194_MOESM1_ESM.pdf]

Supplemental Methods

Quality Control of Methylation Data

In the WHI cohort data, Probes on the X and Y chromosomes (11091 probes), probes with detection p-values > 0.01 in >10% of samples, and samples with detection p-values > 0.01 in >1% of probes were excluded (probes excluded prior to analysis). Probe signals showing multiple clusters, which tend to occur when signals are driven by an underlying SNP or genetic variant, were identified using the gaphunter function in the *minfi* package and removed (54636 probes).

In the ARIC cohort, positive and negative controls and sample replicates were included on each 96-well plate assayed. After exclusion of controls, replicates and samples with integrity issues or failed bisulfite conversion, a total of 2841 study participants had HM450K data available for further QC analyses. We removed poor-quality samples with pass rate of <99%, that is, if the sample had at least 1% of CpG sites with detection P-value > 0.01 or missing (N = 37), indicative of lower DNA quality or incomplete bisulfite conversion, and samples with a possible gender mismatch based on evaluation of selected CpG sites on the Y chromosome (N = 2), leaving a total of 2802 samples available for analysis. At the target level, we flagged poor-quality CpG sites with average detection P-value of >0.01 and calculated the percentage of samples having detection P-value of >0.01 for each autosomal and X chromosome CpG site. There were 9399 autosomal and X chromosomal markers where >1% of samples showed detection P-value of >0.01, and these sites were excluded. In addition, we filtered 370 CpG sites on the Y chromosome with average detection P-value of >0.01, leaving a total of 473788 CpG sites for analysis. After quality control, 428278 probes remained in both analyses and were examined.

Power Calculation

Using the method of Liu and Hwang[50], we tested several scenarios to ensure adequate power to identify the minimum effect size (r^2) and achieve the desired power (>80%) with a FDR q-value<0.05 for 450,000 tests (**Supplemental Table 6**). We tested the minimum effect size for the above aims with a total sample of ~3500 to account for any exclusions. We had >80% power to detect methylation changes associated with the interaction between metabolic health and BMI with an r^2 detecting effects as low as 0.3% of the variance in methylation. Interaction effects are often subtle, but this analysis shows we had power to detect very subtle effects. In the replication analysis, we calculated the minimum effect size for replicating 10, 50 or 1000 CpG sites correcting for multiple testing using Bonferroni correction (0.05/# of tests). We will be able to detect effects if as low as 1% of the variance in methylation can be explained by the interaction.

Supplemental Tables

Table 1. Coefficients and directions of effect in metabolically healthy vs metabolically unhealthy for every one unit increase in BMI in significant sites

CpG site	Coefficient Metabolically Healthy	Coefficient Metabolically Unhealthy	Metabolically Healthy	Metabolically Unhealthy
cg00868074	-0.00038	0.000164	-	+
cg02851049	-8.49E-06	-5.4E-05	-	-
cg05441596	3.48E-05	-5.2E-06	+	-
cg06344952	5.91E-05	-5.2E-05	+	-
cg07226317	3.12E-05	-2.4E-05	+	-
cg08082299	-1.49E-05	-8E-05	-	-
cg10057841	3.25E-05	-5.1E-05	+	-
cg11553983	3.50E-05	-3.3E-06	+	-
cg15062225	8.86E-05	-5.2E-05	+	-
cg16461485	8.27E-05	2.51E-05	+	+
cg16543390	3.05E-05	0.000102	+	+
cg18298785	-2.99E-07	-6.6E-05	-	-
cg18989722	-5.73E-06	-5.3E-05	-	-
cg19572849	-1.67E-05	-6.9E-05	-	-
cg20210586	1.38E-05	-7.4E-05	+	-
cg21880445	-3.03E-05	-9.3E-05	-	-
cg22076143	-1.47E-06	-5.5E-05	-	+
cg24460625	2.29E-06	-6.5E-05	+	-
cg24720717	-7.25E-06	8.54E-05	-	+
cg24827562	9.27E-06	-5E-05	+	-
cg26206680	-7.74E-06	-1.6E-05	-	-
cg27004639	4.21E-05	0.000103	+	+

Table 2. Replication of significant sites in the MESA cohort

CpG Site	T.statistic	P.value	Holm.sig	FDR	Discovery Direction of Effect
cg19572849	0.551567	0.581494	FALSE	0.830705	-
cg18298785	-0.70114	0.483547	FALSE	0.743918	-
cg27004639	0.9877	0.323782	FALSE	0.693479	+
cg11553983	-0.21812	0.827428	FALSE	0.97337	-
cg20210586	-0.10473	0.916631	FALSE	0.97337	-
cg24827562	-1.07273	0.283915	FALSE	0.693479	-
cg26206680	0.092298	0.926499	FALSE	0.97337	-
cg10057841	0.364646	0.715531	FALSE	0.954042	-
cg22076143	0.874629	0.3822	FALSE	0.693479	-
cg24460625	-1.36572	0.172646	FALSE	0.693479	-
cg08082299	1.122892	0.262028	FALSE	0.693479	-
cg05441596	-0.04166	0.966786	FALSE	0.97337	-
cg02851049	-1.71765	0.086487	FALSE	0.693479	-
cg16543390	0.88978	0.374016	FALSE	0.693479	+
cg00868074	0.813911	0.416087	FALSE	0.693479	+
cg06344952	0.033399	0.97337	FALSE	0.97337	-
cg18989722	-2.04684	0.041201	FALSE	0.693479	-
cg16461485	0.920949	0.357526	FALSE	0.693479	-
cg21880445	-0.95512	0.339982	FALSE	0.693479	-
cg15062225	-1.10901	0.267963	FALSE	0.693479	-

Table 3. Association between methylation in significant sites and incident myocardial infarction

CpG site	Hazard Ratio	Lower Limit	Upper Limit	Z-score	p-value	FDR q-value
cg00868074	1.002611	0.986327	1.019165	0.312138	0.754935	0.830429
cg02851049	0.897052	0.812154	0.990825	-2.14171	0.032217	0.249169
cg05441596	1.026875	0.935722	1.126908	0.559177	0.576041	0.722072
cg06344952	0.970311	0.895086	1.051858	-0.73202	0.464157	0.693788
cg07226317	1.030871	0.948716	1.12014	0.717546	0.473038	0.693788
cg08082299	0.971861	0.934026	1.011229	-1.40884	0.158883	0.488638
cg10057841	1.030443	0.966818	1.098256	0.922249	0.356399	0.604376
cg11553983	1.044131	0.95348	1.1434	0.931961	0.351357	0.604376
cg15062225	0.972661	0.944136	1.002048	-1.8253	0.067955	0.249169
cg16461485	0.941783	0.888621	0.998125	-2.0233	0.043042	0.249169
cg16543390	1.045665	0.9971	1.096596	1.840311	0.065723	0.249169
cg18298785	0.997793	0.920392	1.081704	-0.05363	0.957233	0.957233
cg18989722	1.043733	0.952845	1.143292	0.920846	0.357131	0.604376
cg19572849	0.994284	0.933883	1.058591	-0.17927	0.857724	0.898568
cg20210586	1.094975	1.003004	1.19538	2.027009	0.042661	0.249169
cg21880445	0.973924	0.884499	1.07239	-0.5377	0.590786	0.722072
cg22076143	0.983697	0.907202	1.066643	-0.39796	0.690658	0.799709
cg24460625	0.970914	0.928067	1.015739	-1.28184	0.199898	0.488638
cg24720717	1.0347	0.915568	1.169333	0.546571	0.584673	0.722072
cg24827562	0.932045	0.840205	1.033923	-1.32967	0.183626	0.488638
cg26206680	1.067812	0.946587	1.204561	1.067173	0.285894	0.604376
cg27004639	1.065113	0.997925	1.136825	1.89752	0.057759	0.249169

Table 4. Relevant CpG site associations with mRNA transcripts from the MESA and GTP cohort

CpG.probe	exp.Probe	exp.probe.chrm	exp.probe.start	exp.probe.stop	exp.probe.strand	annot.gene	in_dist	distance	status	other_gene	distance.other.gene	p.val	T.stat	beta	beta.sd	Cohort
cg18989722	ILMN_1665100	9	1.25E+08	1.25E+08	+	PTGS1	NA	NA	TRANS	NA	NA	2.41E-06	4.739083	0.038621	0.008149453	MESA
cg16461485	ILMN_1759075	17	16842449	16842498	-	TNFRSF13B	NA	NA	TRANS	NA	NA	5.87E-06	-4.60758689503	-0.05542014	1.20E-02	GTP

Table 5. Correlation between effect sizes in the significant 22 sites between primary analysis and results when leaving out one clinical parameter

Clinical Parameter	Effect Estimate r
Glucose	0.9525753
Blood Pressure	0.9767315
Waist Circumference	0.9751579
Triglycerides	0.9565201
HDL-cholesterol	0.8994886

Table 6. Minimum effect size for identifying significant sites in discovery and replication analyses

# of DM CpG sites	10	50	1000
Discovery r^2	0.0048	0.0041	0.0032
Replication r^2	0.0105	0.013	0.0187
DM=differentially methylated			

Supplemental Figure 1.

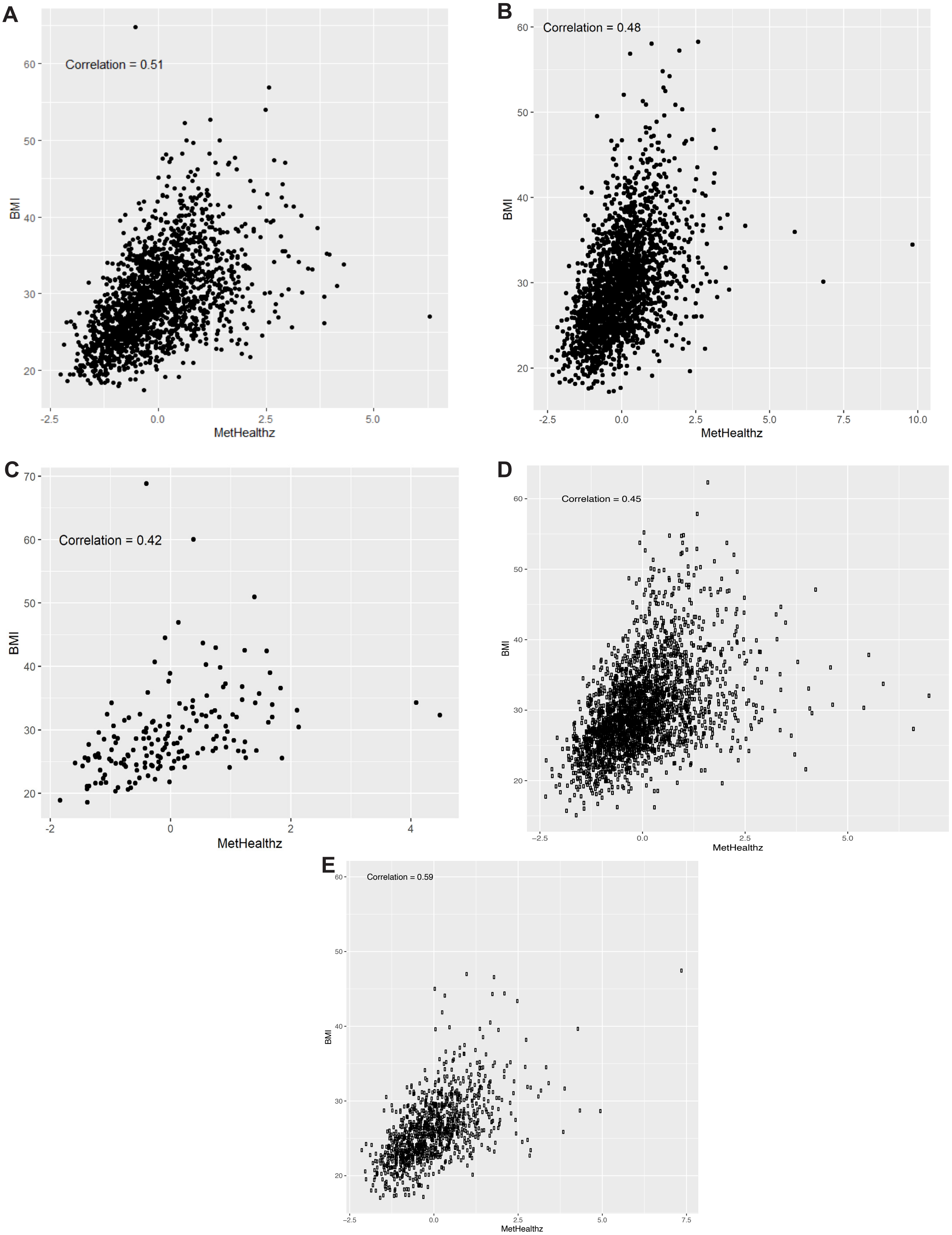

Supplemental Figure 1. Correlation between body mass index (BMI) and metabolic health Z-score (MHZ) in WHI EMPC (A), WHI BAA23 (B), WHI AS311 (C), ARIC AA (D), and ARIC EA (E).

Supplemental Figure 2.

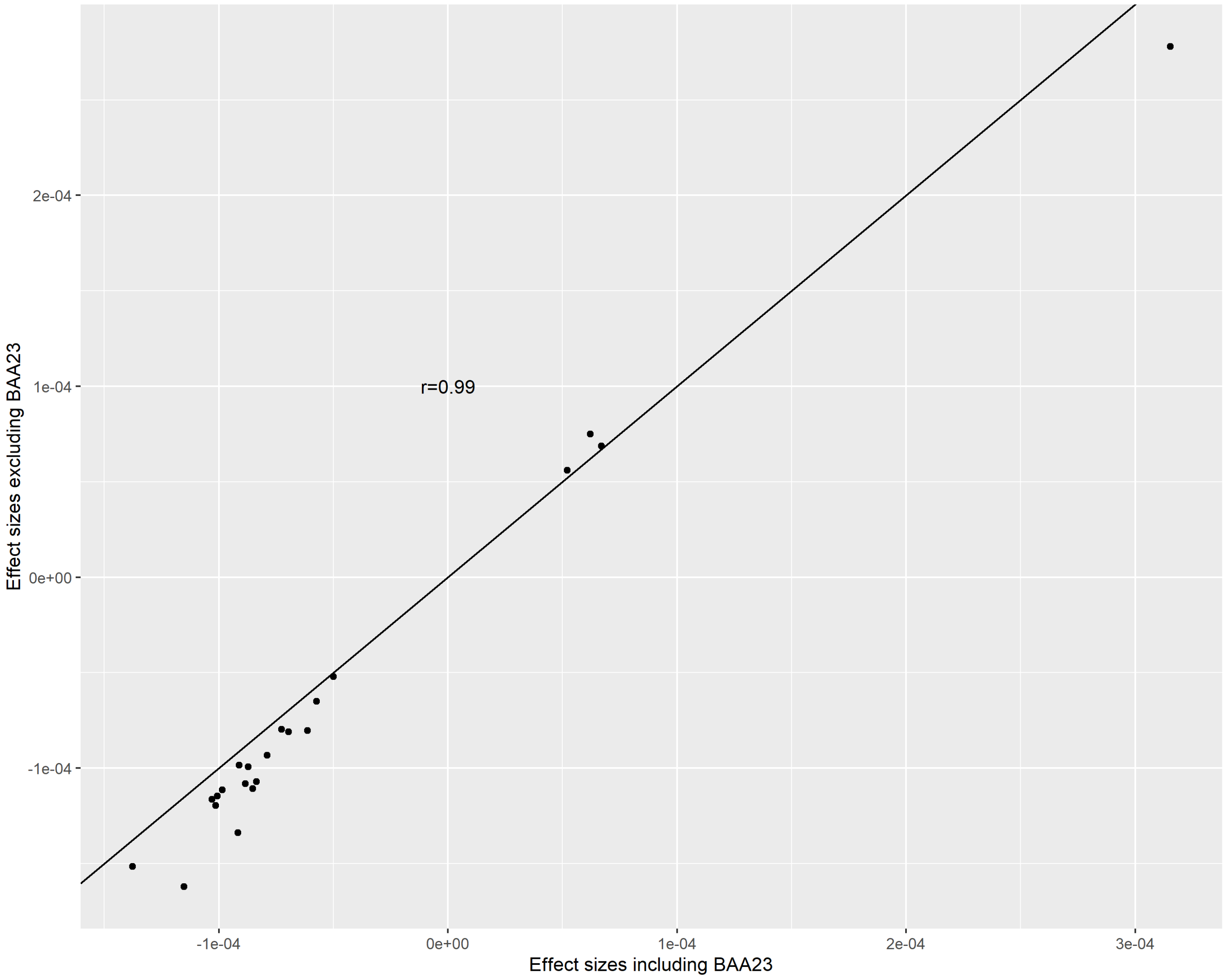

Supplemental Figure 2. Comparison of effect sizes when excluding BAA23 and including BAA23

Supplemental Figure 3.

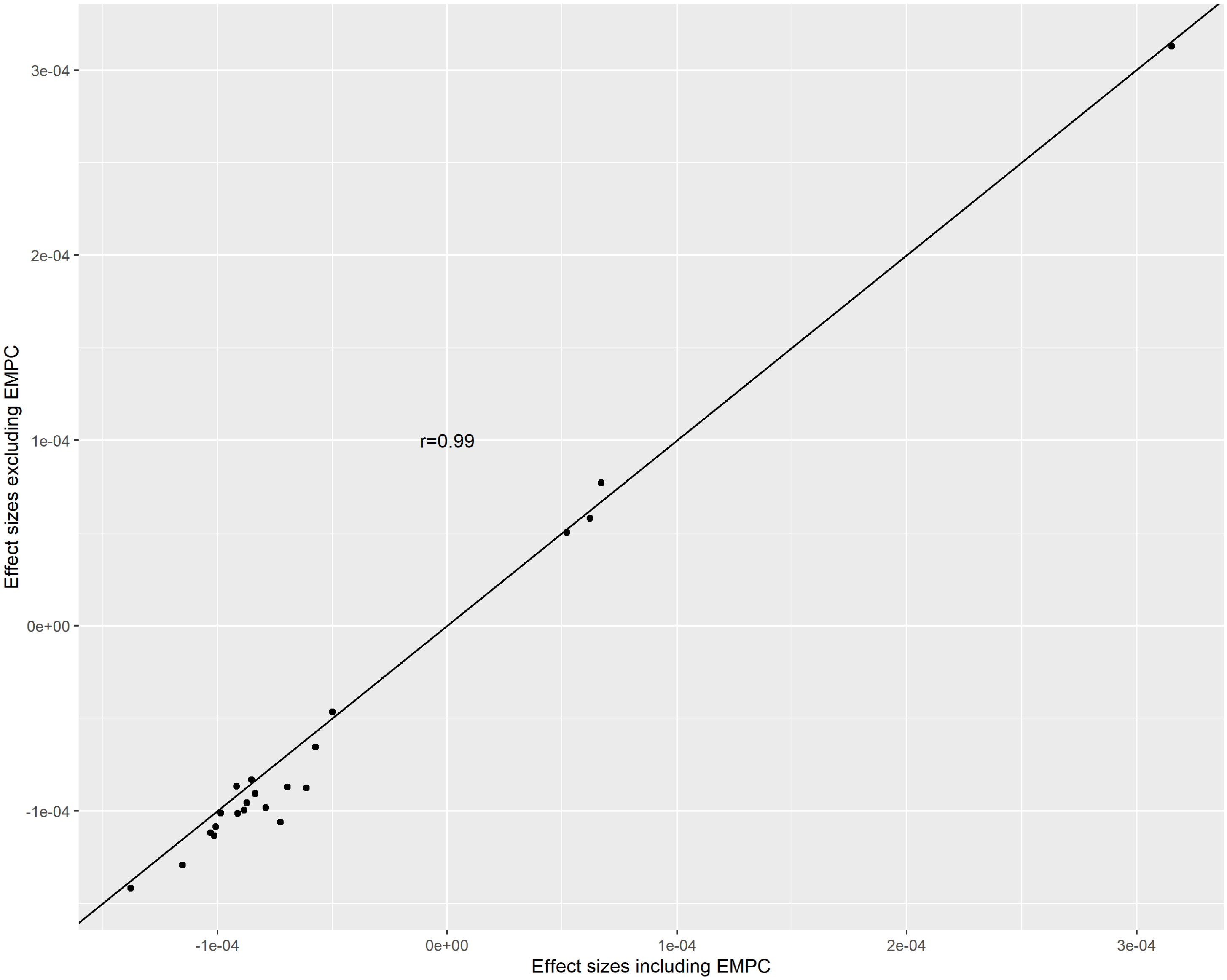

Supplemental Figure 3. Comparison of effect sizes when excluding EMPC and including EMPC

Supplemental Figure 4.

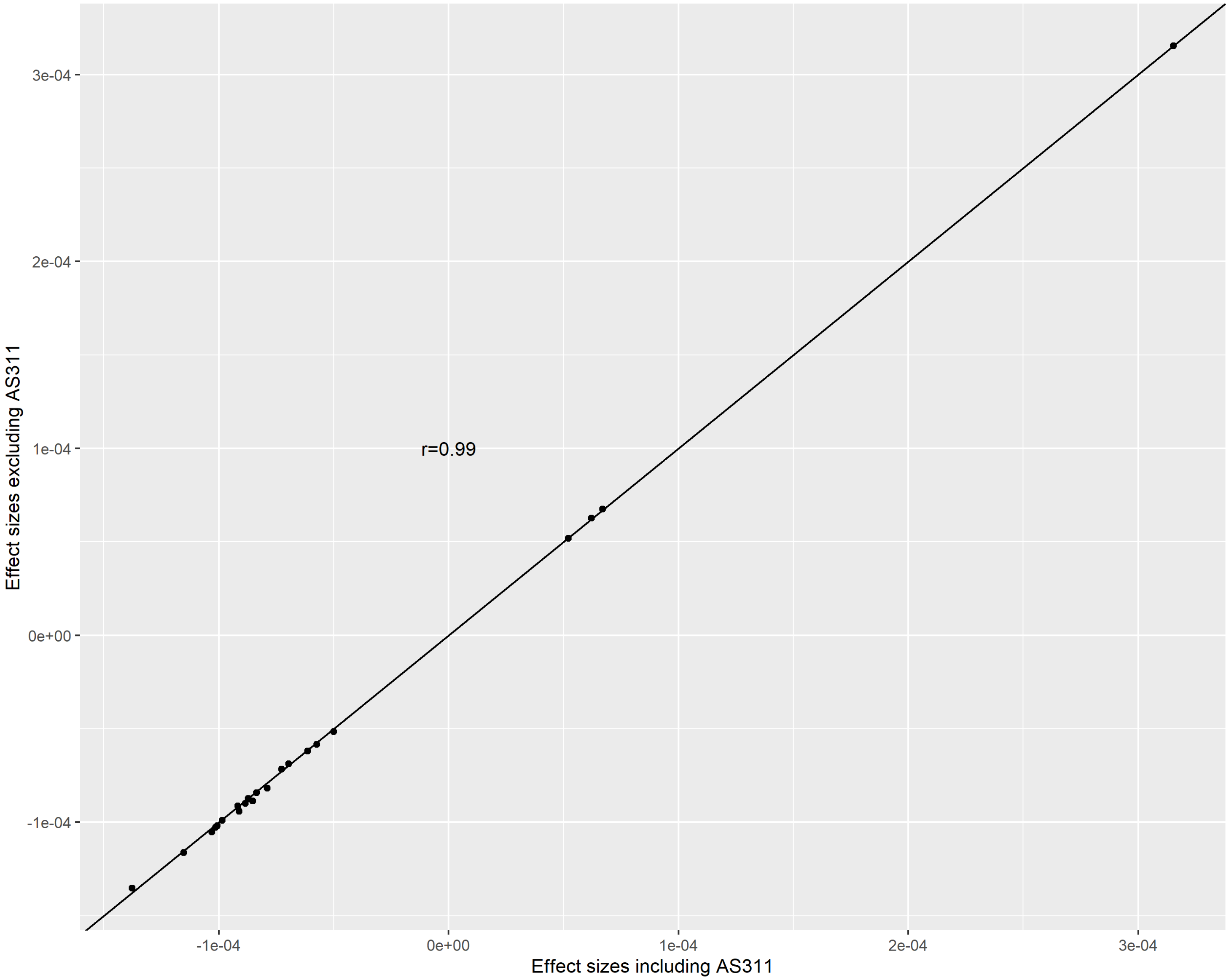

Supplemental Figure 4. Comparison of effect sizes when excluding AS311 and including AS311

Supplemental Figure 5.

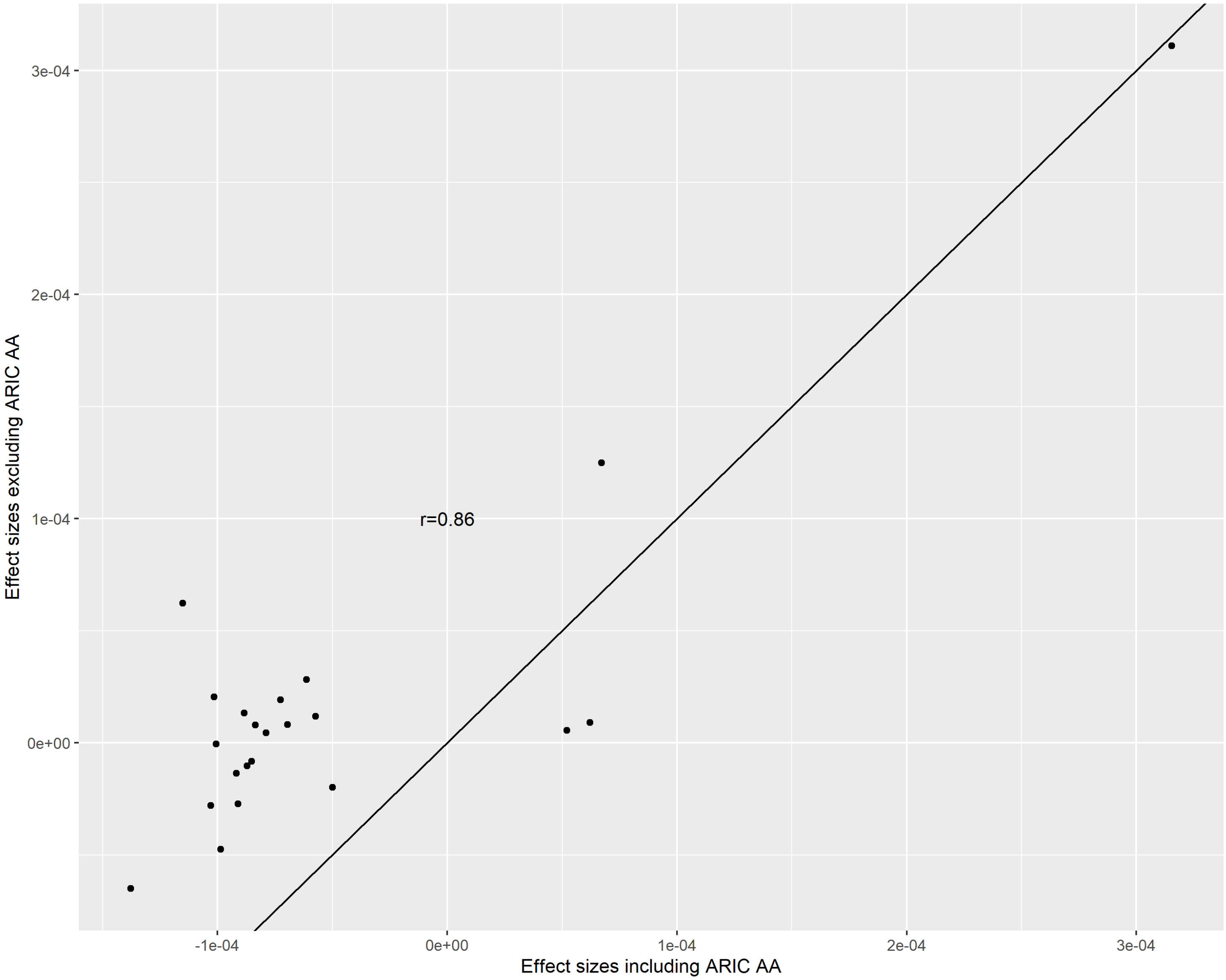

Supplemental Figure 5. Comparison of effect sizes when excluding ARIC AA and including ARIC AA.

Supplemental Figure 6.

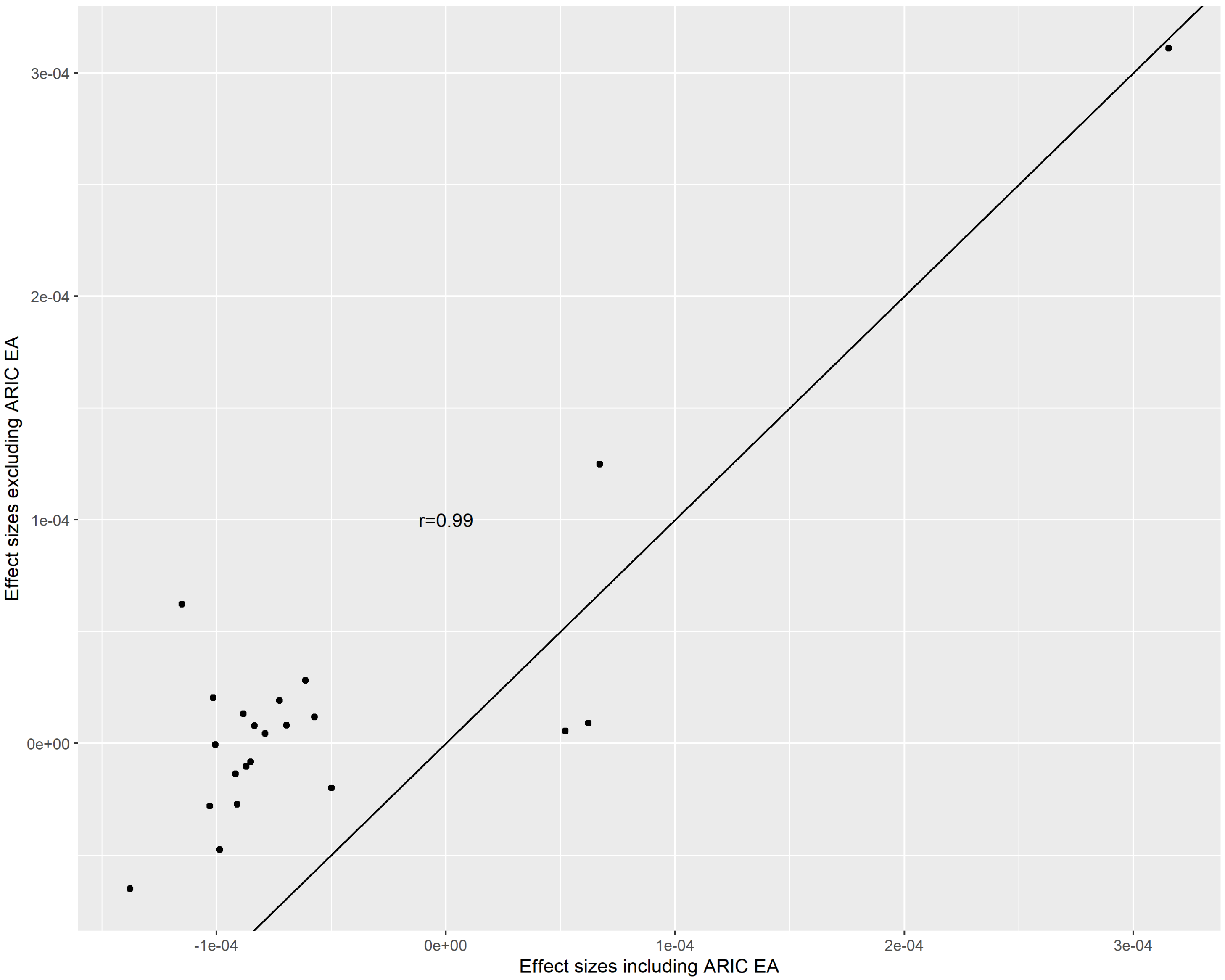

Supplemental Figure 6. Comparison of effect sizes when excluding ARIC EA and including ARIC EA.

Supplemental Figure 7.

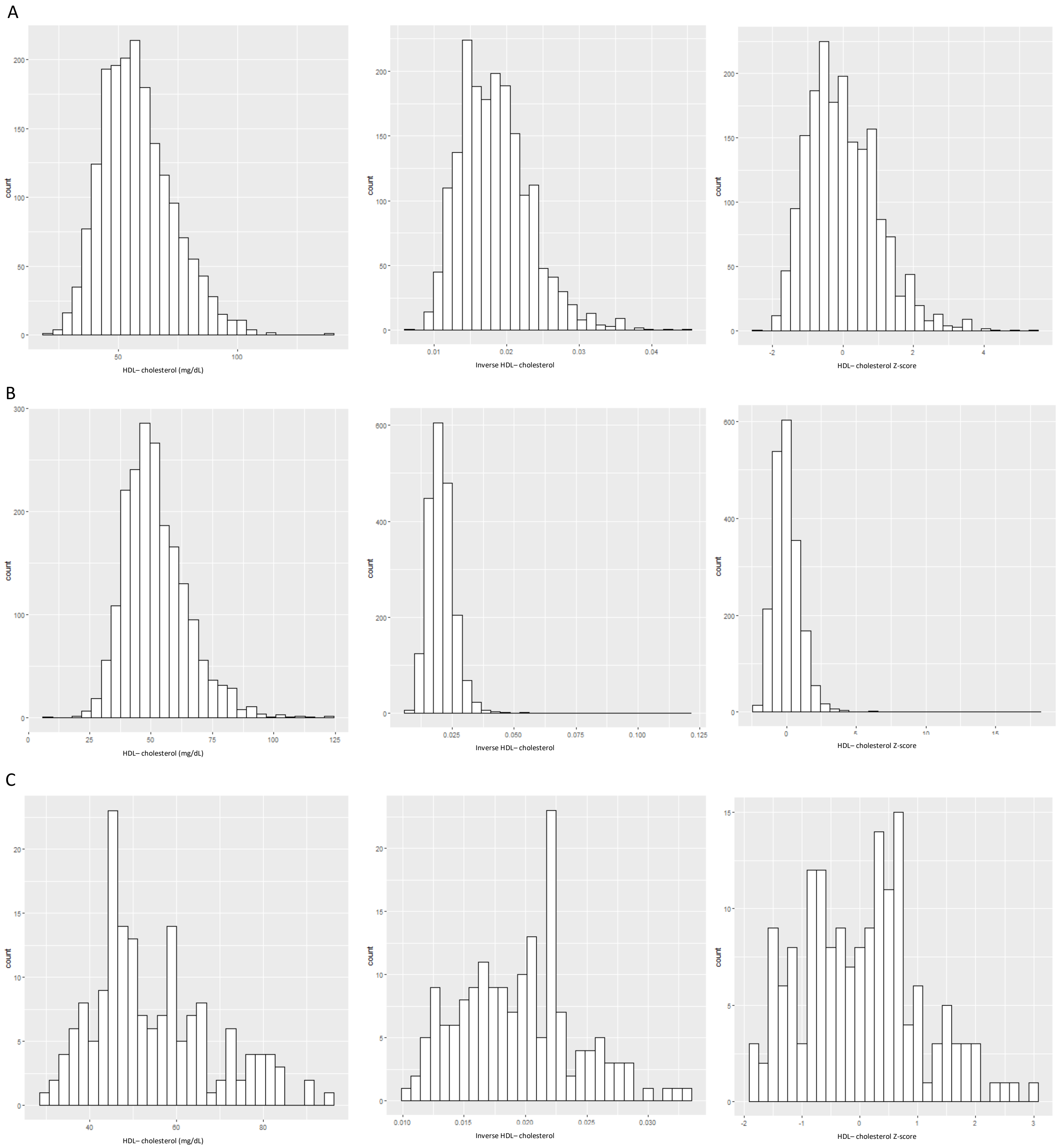

Supplemental Figure 7. Distribution of HDL-cholesterol, inverse HDL-cholesterol and HDL-cholesterol Z-score by ancillary cohort (Epigenetic mechanisms of particulate matter-mediated cardiovascular disease [EMPC, A], Integrative Genomics for Risk of Coronary Heart Disease and Related Phenotypes in WHI cohort [BAA23, B], Bladder Cancer and Leukocyte Methylation [AS311, C]) in the Women's Health Initiative (WHI)

Supplemental Figure 8.

A

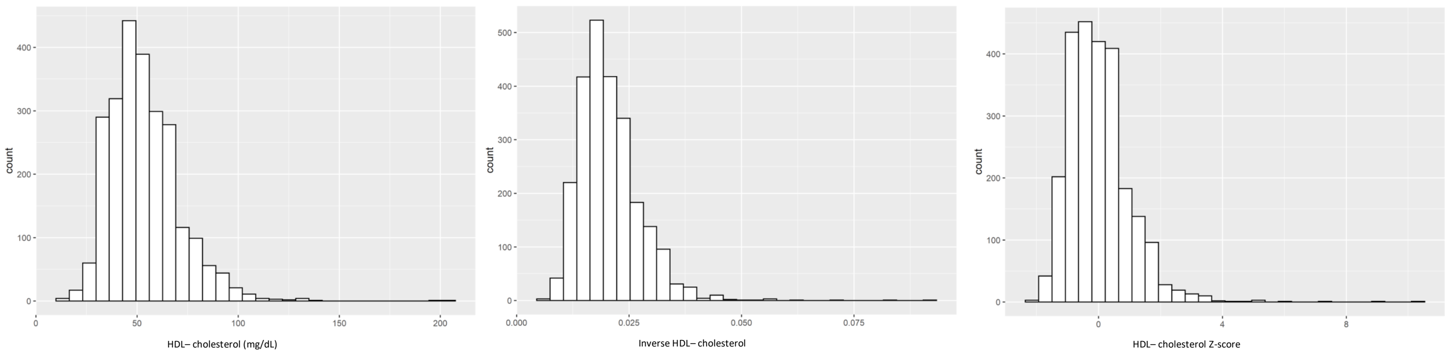

B

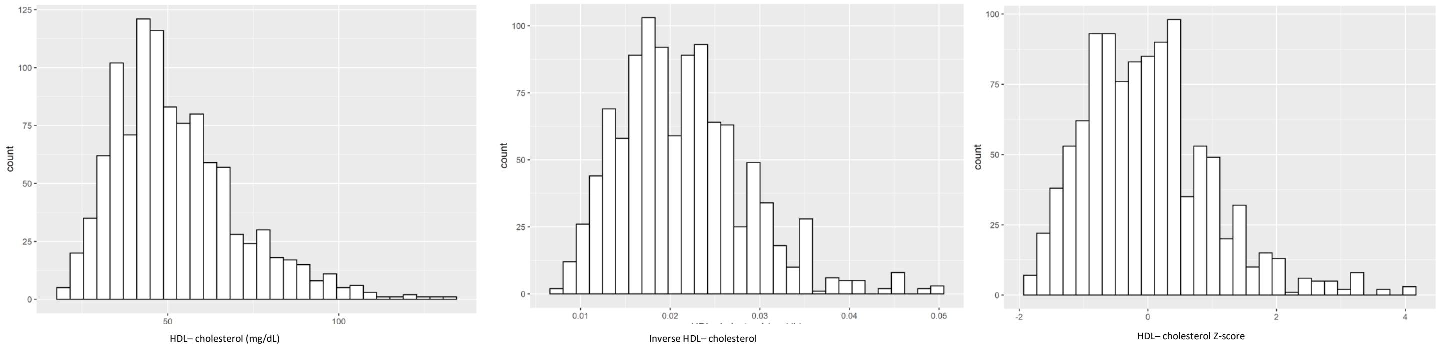

Supplemental Figure 8. Distribution of HDL-cholesterol, inverse HDL-cholesterol and HDL-cholesterol Z-score by race/ethnicity (African Americans [AA, A] and European Americans [EA, B]) in the Atherosclerosis Risk in Communities (ARIC) cohort

Supplemental Figure 9

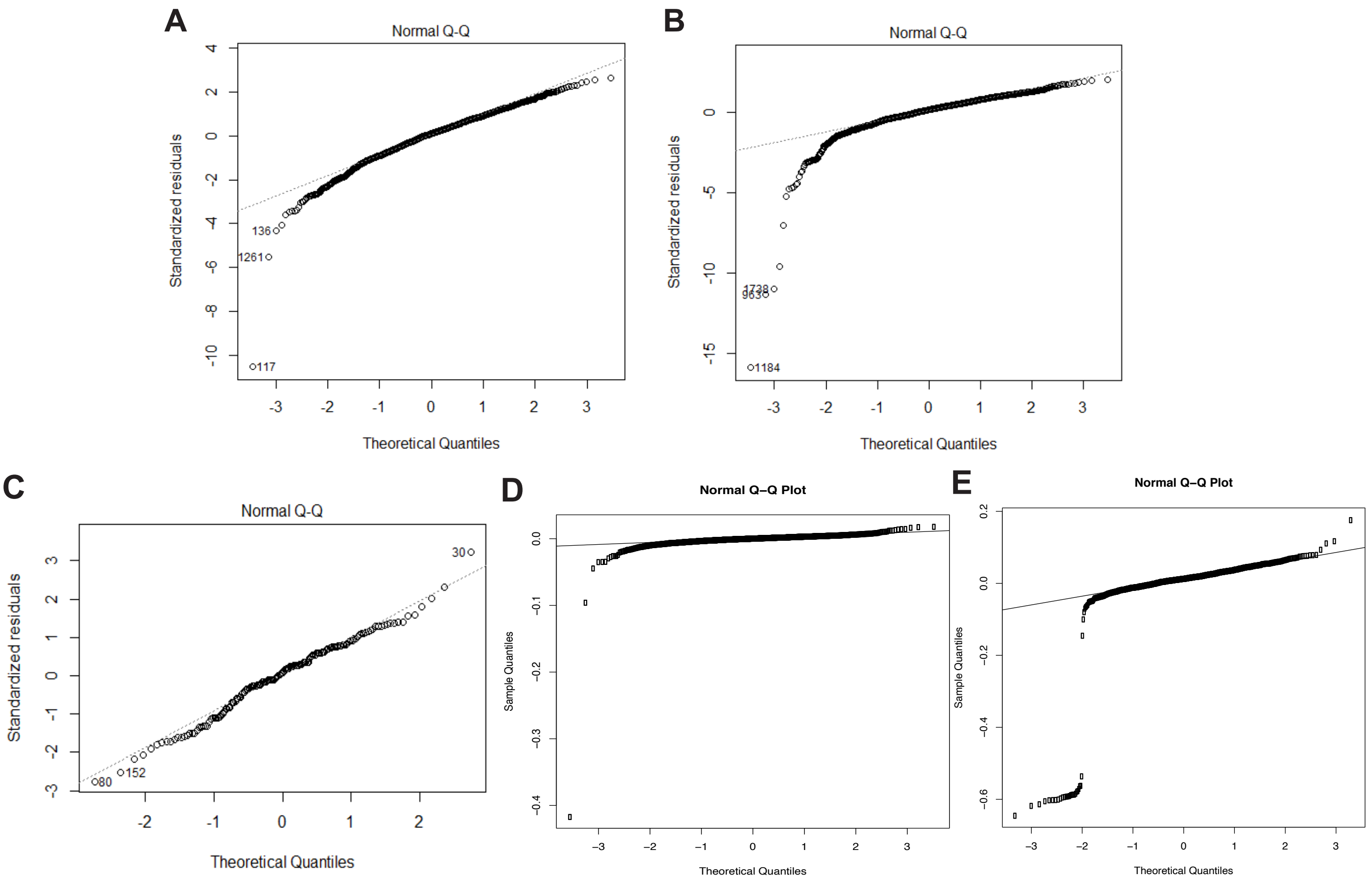

Supplemental Figure 9. QQ plot examining the residuals for cg02851049 in WHI EMPC (A), WHI BAA23 (B), WHI AS311 (C), ARIC AA (D), and ARIC EA (E).

Supplemental Figure 9

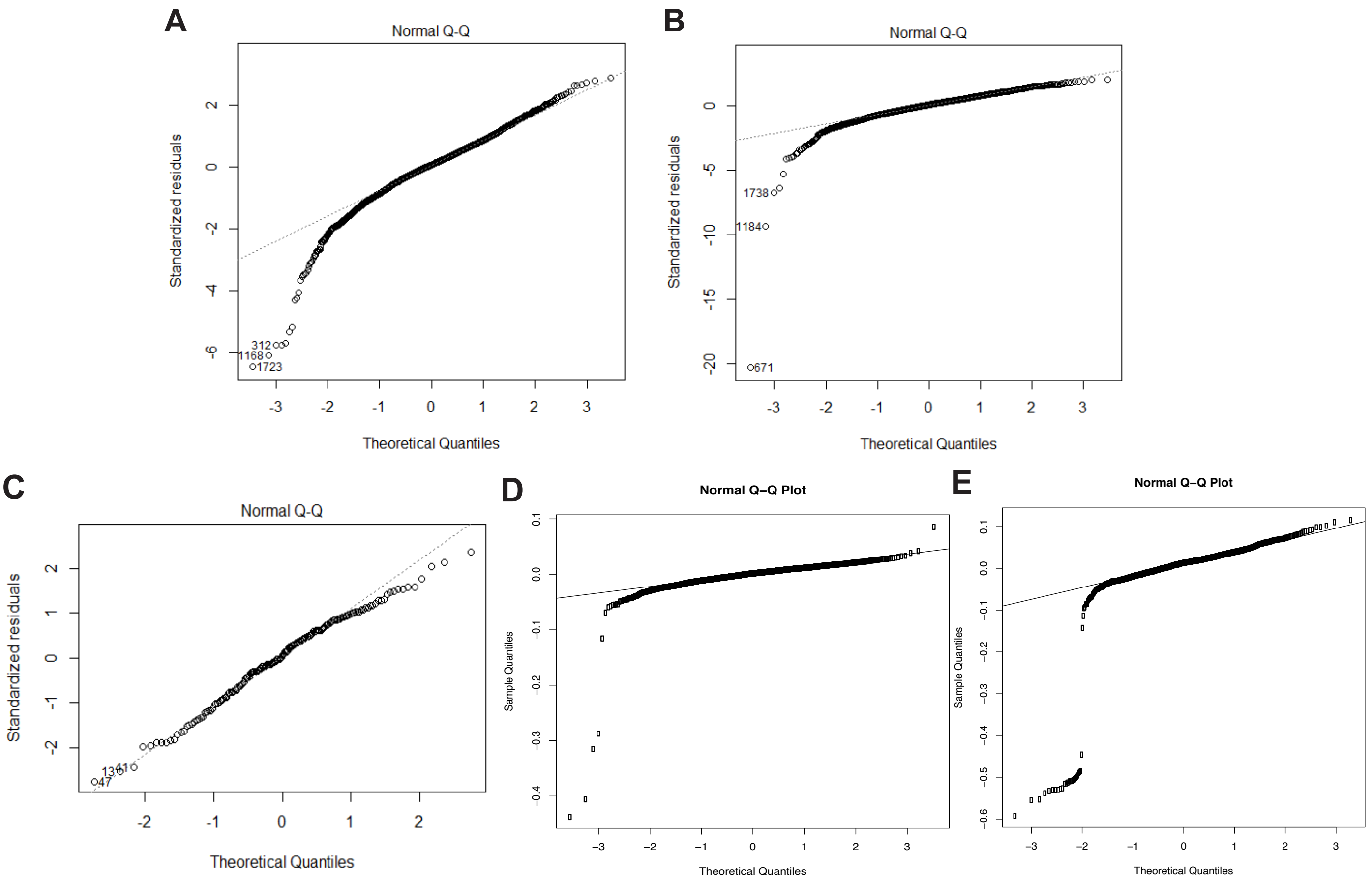

Supplemental Figure 9. QQ plot examining the residuals for cg16461485 in WHI EMPC (A), WHI BAA23 (B), WHI AS311 (C), ARIC AA (D), and ARIC EA (E).

Supplemental Figure 10

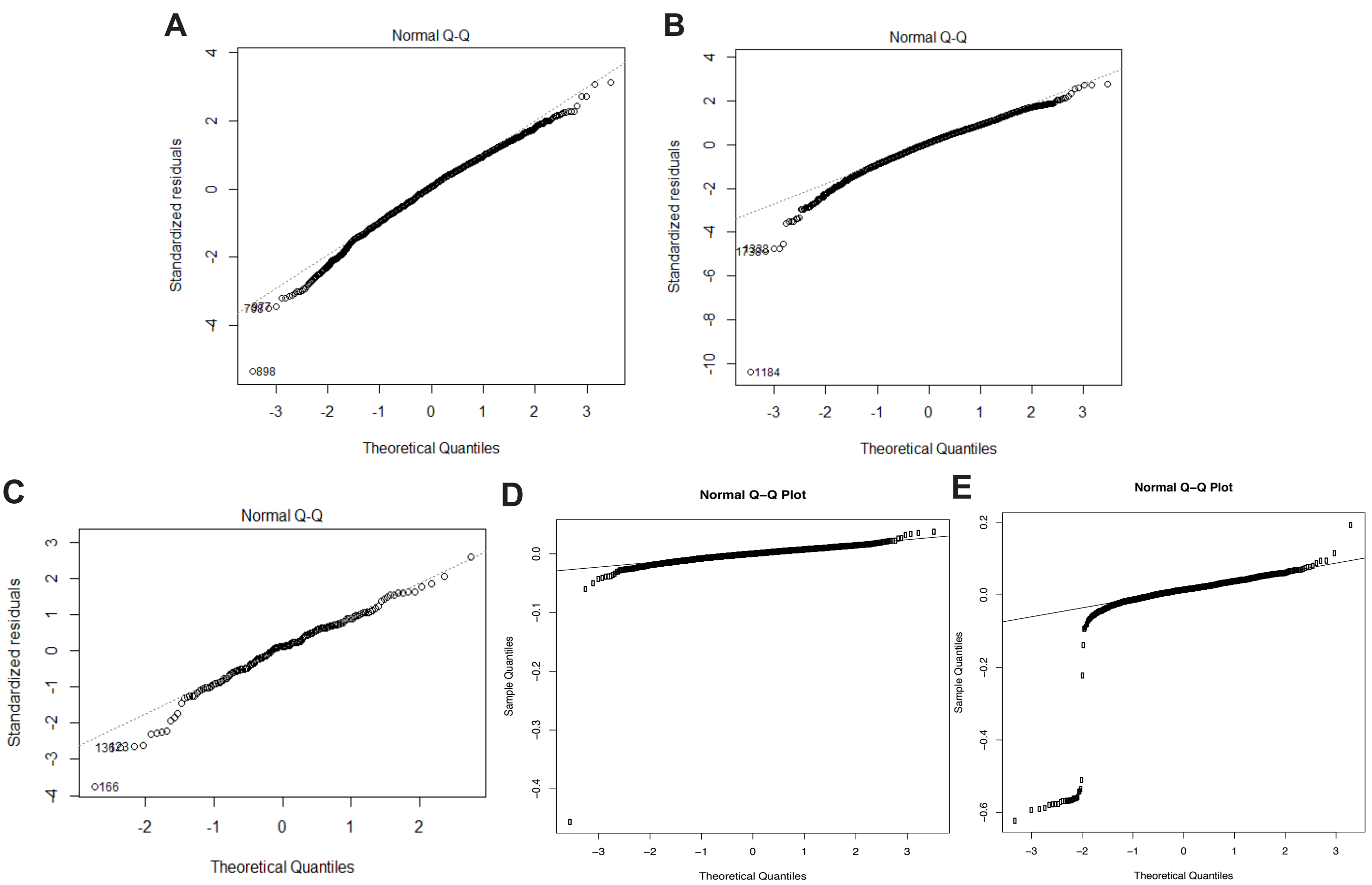

Supplemental Figure 10. QQ plot examining the residuals for cg20210586 in WHI EMPC (A), WHI BAA23 (B), WHI AS311 (C), ARIC AA (D), and ARIC EA (E).

Supplemental Figure 11

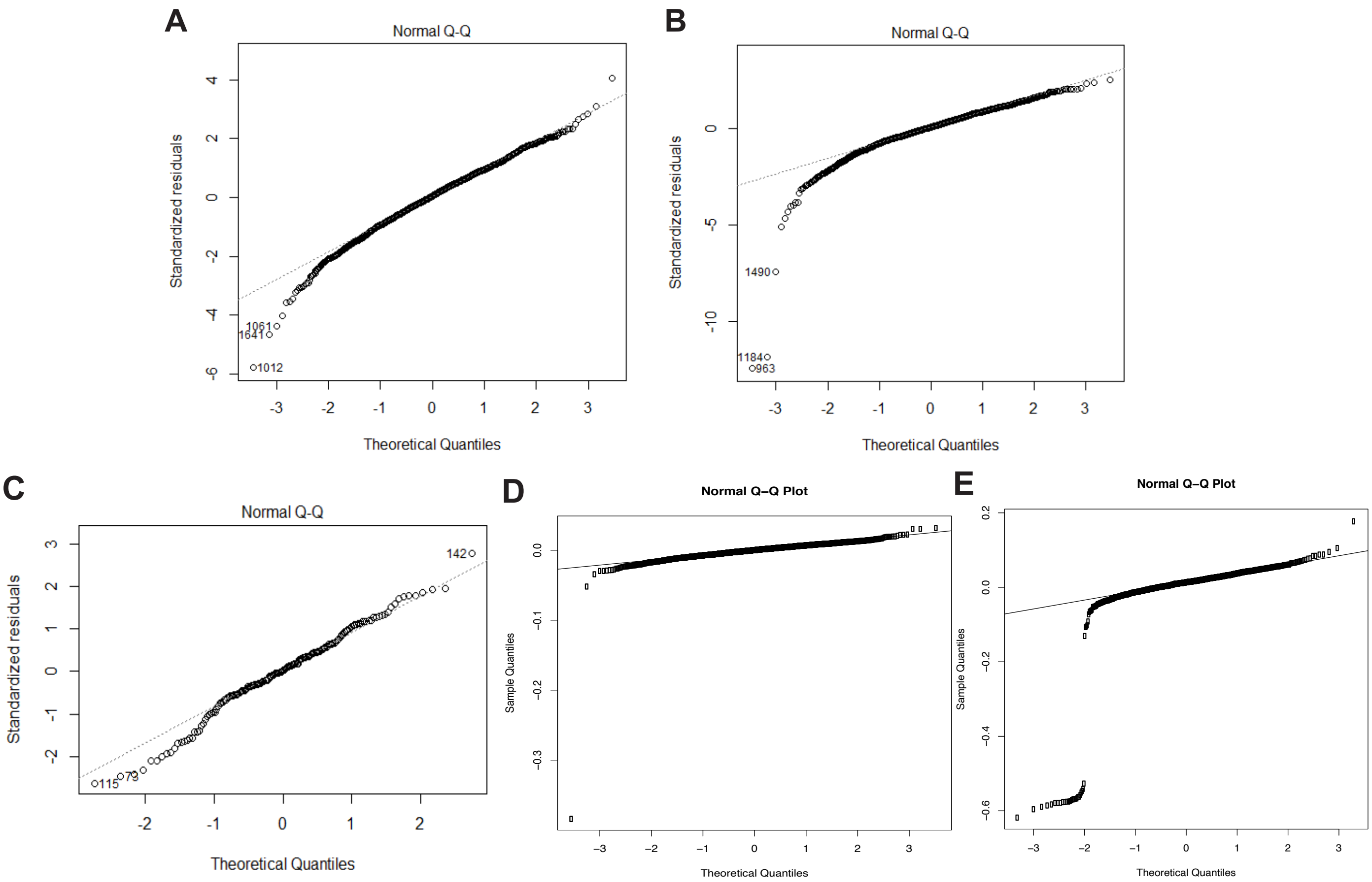

Supplemental Figure 11. QQ plot examining the residuals for cg18989722 in WHI EMPC (A), WHI BAA23 (B), WHI AS311 (C), ARIC AA (D), and ARIC EA (E).

Supplemental Figure 12

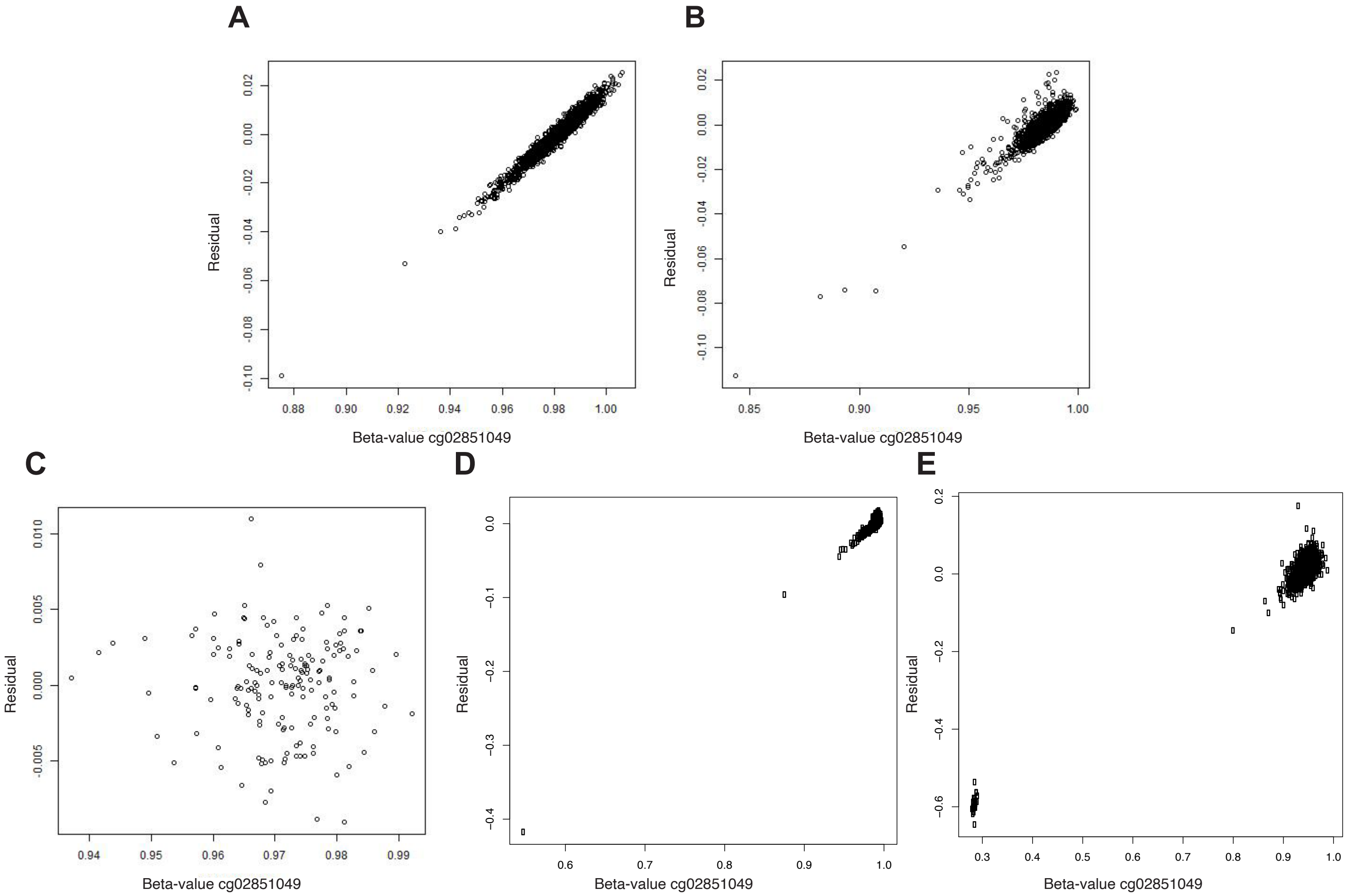

Supplemental Figure 12. Examining residuals plot in cg02851049 in WHI EMPC (A), WHI BAA23 (B), WHI AS311 (C), ARIC AA (D), and ARIC EA (E).

Supplemental Figure 13

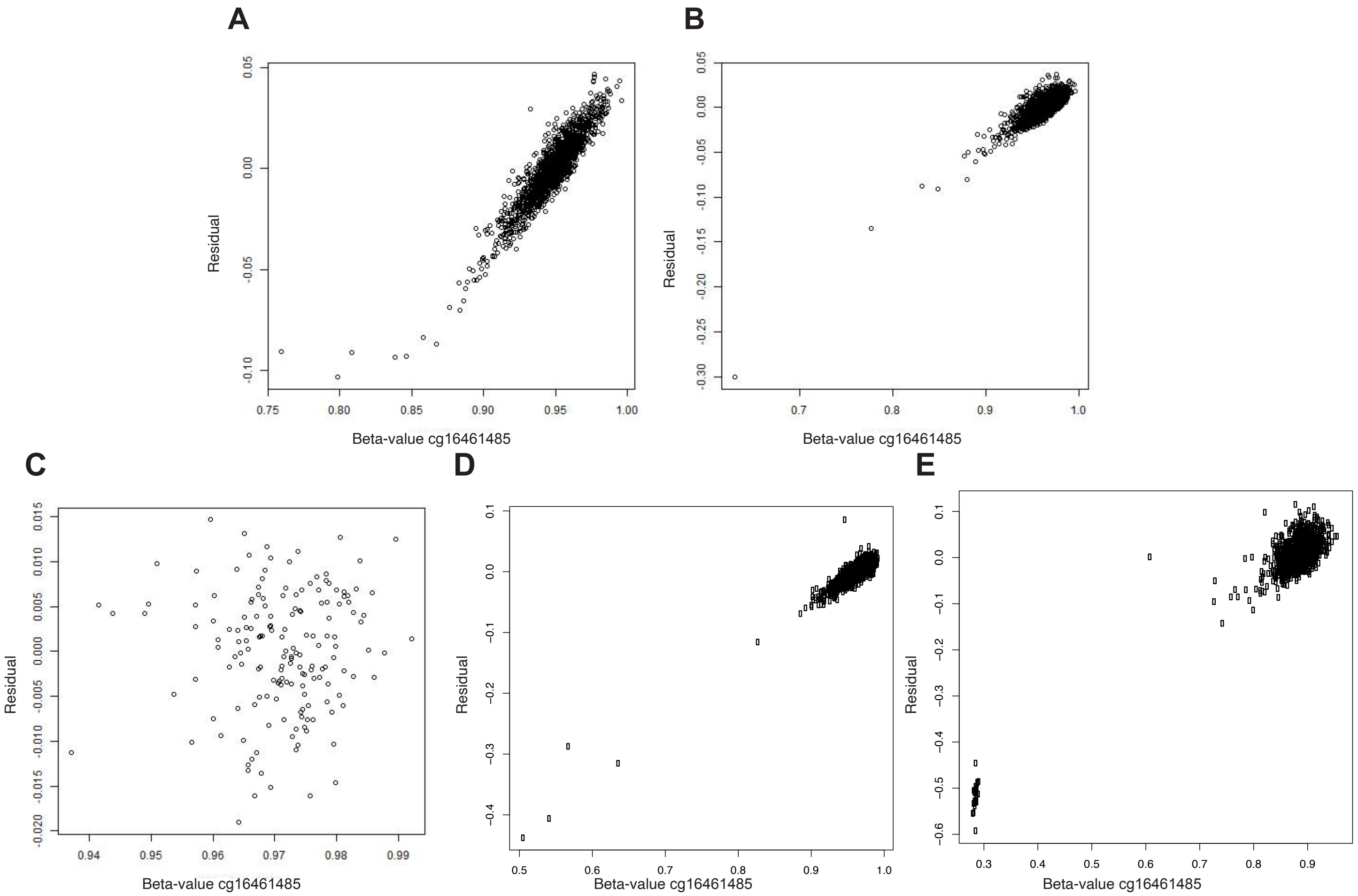

Supplemental Figure 13. Examining residuals plot in cg16461485 in WHI EMPC (A), WHI BAA23 (B), WHI AS311 (C), ARIC AA (D), and ARIC EA (E).

Supplemental Figure 14

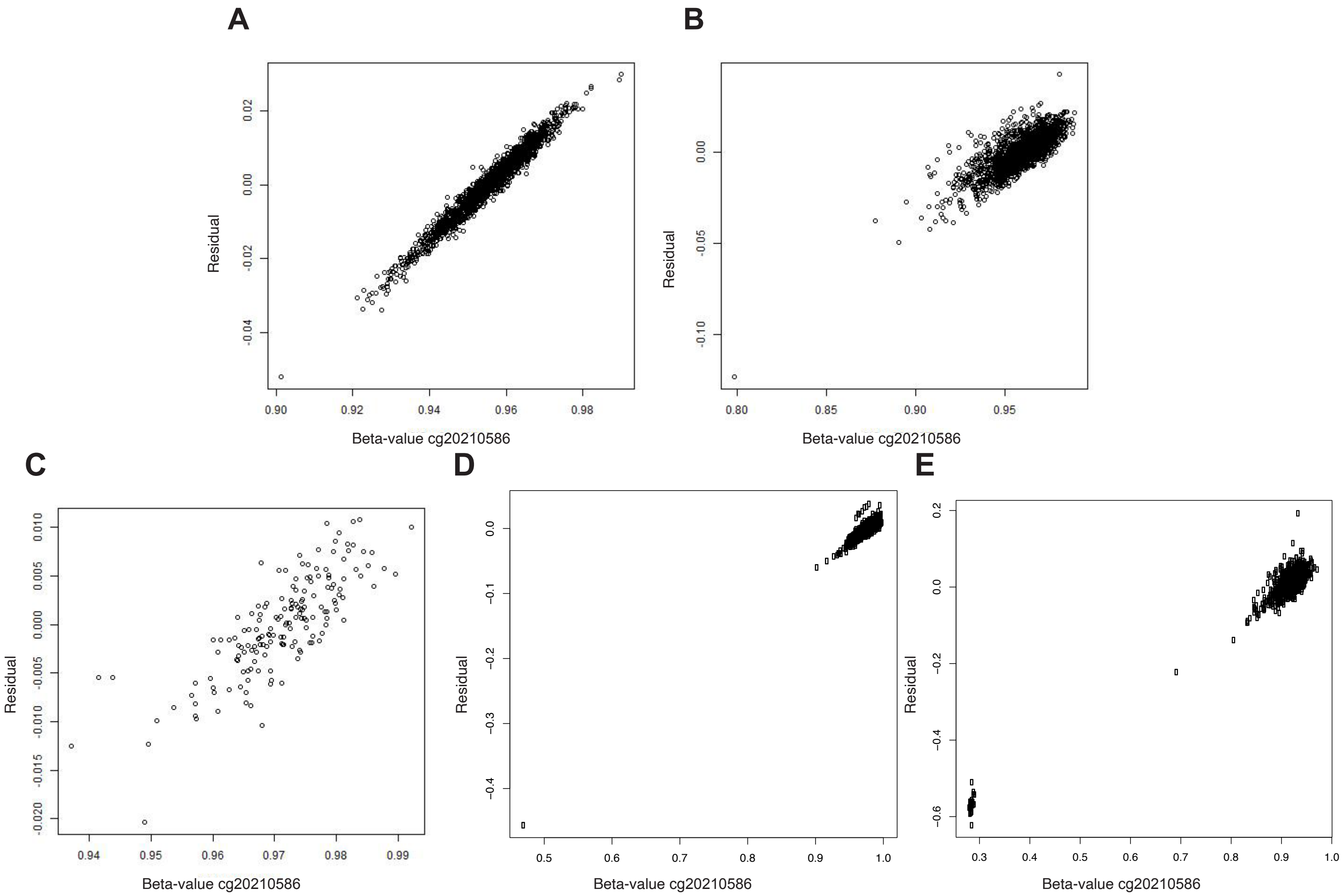

Supplemental Figure 14. Examining residuals plot in cg20210586 in WHI EMPC (A), WHI BAA23 (B), WHI AS311 (C), ARIC AA (D), and ARIC EA (E).

Supplemental Figure 15

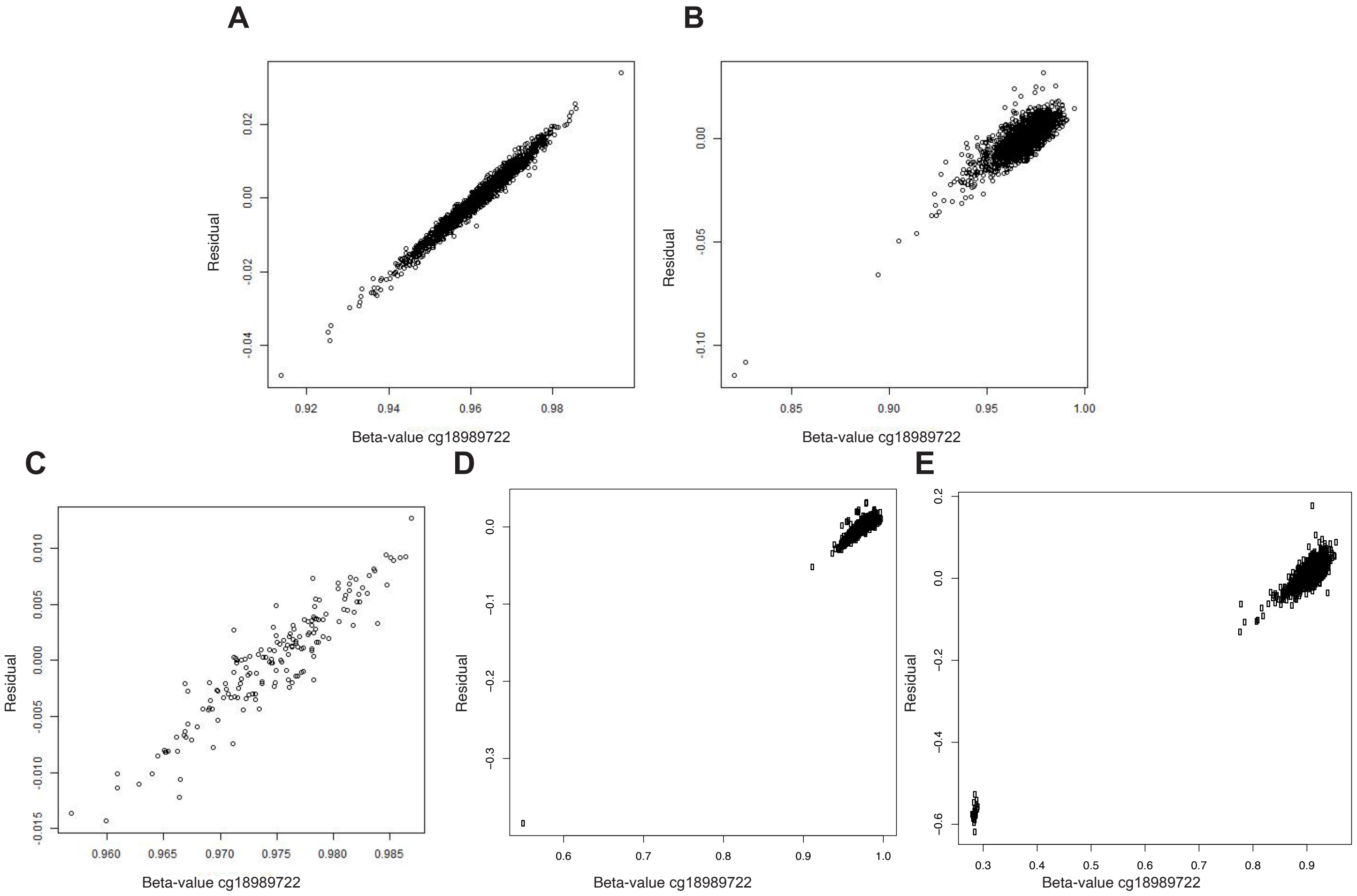

Supplemental Figure 15. Examining residuals plot in cg18989722 in WHI EMPC (A), WHI BAA23 (B), WHI AS311 (C), ARIC AA (D), and ARIC EA (E).
